# A 3D Fiber‐Hydrogel Based Non‐Viral Gene Delivery Platform Reveals that microRNAs Promote Axon Regeneration and Enhance Functional Recovery Following Spinal Cord Injury

**DOI:** 10.1002/advs.202100805

**Published:** 2021-05-29

**Authors:** Na Zhang, Junquan Lin, Vincent Po Hen Lin, Ulla Milbreta, Jiah Shin Chin, Elaine Guo Yan Chew, Michelle Mulan Lian, Jia Nee Foo, Kunyu Zhang, Wutian Wu, Sing Yian Chew

**Affiliations:** ^1^ School of Chemical and Biomedical Engineering Nanyang Technological University 62 Nanyang Drive Singapore 637459 Singapore; ^2^ Interdisciplinary Graduate School Nanyang Technological University 61 Nanyang Drive Singapore 637335 Singapore; ^3^ Human Genetics Genome Institute of Singapore 60 Biopolis Street Singapore 138672 Singapore; ^4^ Lee Kong Chian School of Medicine Nanyang Technological University 59 Nanyang Drive Singapore 636921 Singapore; ^5^ Guangdong‐Hongkong‐Macau Institute of CNS Regeneration Ministry of Education CNS Regeneration Collaborative Joint Laboratory Jinan University 601 West Huangpu Avenue Guangzhou 510632 P. R. China; ^6^ Re‐Stem Biotechnology Co., Ltd. 1463 Wuzhong Ave Suzhou 330520 P. R. China

**Keywords:** electrospinning, hydrogel, neural tissue engineering, RNA interference, RNA sequencing

## Abstract

Current treatment approaches toward spinal cord injuries (SCI) have mainly focused on overcoming the inhibitory microenvironment that surrounds lesion sites. Unfortunately, the mere modulation of the cell/tissue microenvironment is often insufficient to achieve desired functional recovery. Therefore, stimulating the intrinsic growth ability of injured neurons becomes crucial. MicroRNAs (miRs) play significant roles during axon regeneration by regulating local protein synthesis at growth cones. However, one challenge of using miRs to treat SCI is the lack of efficient delivery approaches. Here, a 3D fiber‐hydrogel scaffold is introduced which can be directly implanted into a spinal cord transected rat. This 3D scaffold consists of aligned electrospun fibers which provide topographical cues to direct axon regeneration, and collagen matrix which enables a sustained delivery of miRs. Correspondingly, treatment with Axon miRs (i.e., a cocktail of miR‐132/miR‐222/miR‐431) significantly enhances axon regeneration. Moreover, administration of Axon miRs along with anti‐inflammatory drug, methylprednisolone, synergistically enhances functional recovery. Additionally, this combined treatment also decreases the expression of pro‐inflammatory genes and enhance gene expressions related to extracellular matrix deposition. Finally, increased Axon miRs dosage with methylprednisolone, significantly promotes functional recovery and remyelination. Altogether, scaffold‐mediated Axon miR treatment with methylprednisolone is a promising therapeutic approach for SCI.

## Graphical abstract

A novel approach of targeting axon local protein synthesis to promote the intrinsic growth ability of neurons by scaffold‐mediated microRNA delivery is introduced. The biofunctional 3D fiber‐hydrogel scaffold provides topographical cues that direct axon regrowth and a novel microRNA cocktail (miR‐132/miR‐222/miR‐431), which in the presence of methylprednisolone, enhances both nerve regeneration and functional recovery after spinal cord injury.

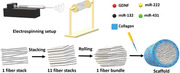

## 1 Introduction

Spinal cord injuries (SCI) result in devastating outcomes of paralysis and functional impairment and are the major causes of morbidity and mortality, particularly in young adults and children.^[^ ^1^ ^]^ Both intrinsic growth ability of neurons and the microenvironment that surrounds cells/tissues control the process of nerve (axon) regeneration after injuries.^[^ ^2^, ^3^ ^]^ However, current SCI treatment approaches mainly focus on overcoming the inhibitory microenvironment that is present after nerve injuries. Results available to date reflect sub‐optimal recovery of function in patients. An alternative is to recognize that mature neurons have diminished intrinsic regeneration capability, which is a major cause of regeneration failure. Therefore, the mere modulation of the microenvironment may be insufficient to achieve the desired regeneration outcomes^[^ ^4^ ^]^ and stimulating the intrinsic growth ability of mature neurons becomes crucial.

Instead of focusing only on delivering inductive factors to stimulate nerve regeneration, a combinatorial approach may be to silence inhibitory genes involved in axon regeneration by RNA‐interference (RNAi). As small non‐coding RNAs, microRNAs (miRs) are crucial gene/protein regulators that actively maintain the central nervous system (CNS).^[^ ^5^ ^]^ In neurons, miRs play important roles in mediating their development,^[^ ^6^, ^7^ ^]^ forming functional circuitry,^[^ ^8^ ^]^ providing navigational guidance,^[^ ^9^ ^]^ as well as, modulating axonal outgrowth and branching.^[^ ^10^ ^]^ In particular, when miRs (i.e., miR‐132,^[^ ^11^ ^]^ miR‐222,^[^ ^12^ ^]^ miR‐431^[^ ^13^ ^]^) were overexpressed in injured neurons, enhanced axon regeneration occurred from the growth cones.^[^ ^12^, ^13^, ^14^, ^15^ ^]^ These miRs either enhance pathways that regulate neurogenesis and axon growth^[^ ^13^ ^]^ or directly remove molecular brakes^[^ ^12^ ^]^ that prevent regrowth. Specifically, Ras GTPase, p120RasGAP (Rasa1), which is involved in cytoskeletal regulation, is targeted by miR‐132.^[^ ^15^ ^]^ In addition, Wnt signaling, which is significant for neurogenesis and axon growth, is regulated by miR‐431 as miR‐431 decreases the expression of a Wnt antagonist, Kremen1.^[^ ^13^ ^]^ Finally, miR‐222 targets PTEN,^[^ ^12^ ^]^ an inhibitor of the PI3K pathway that is important to central axon growth.

Importantly, the concept of “more is good” may be flawed where miRs are concerned and the combination used must be properly tested in order to derive the most effective outcomes. While we have screened various combinations of miRs to derive the optimal combination that comprises of miR‐132, miR‐222, and miR‐431 (a.k.a. Axon miRs), and we further demonstrated that Axon miRs promoted axonal regeneration both in vitro and in vivo,^[^ ^16^ ^]^ how Axon miRs orchestrate this regenerative process remains unknown. Moreover, there is also a lack of information regarding the types of regenerated axons and whether such miR treatment will contribute to functional recovery at later time points. Consequently, attempts to answer these questions will provide greater insights for future miR‐based treatments for SCI.

To address the questions raised above, it is essential to develop an efficient non‐viral miR delivery method which can be applicable to treat SCI. Here, a scaffold‐mediated non‐viral miR delivery approach was established in order to effectively deliver miRs into the transacted rat spinal cord. Specifically, this 3D hybrid fiber‐hydrogel scaffold consists of aligned electrospun fibers which resemble the architecture of the natural microenvironment that surrounds cells, as well as provide aligned topographical cues to direct axon regeneration. Besides that, the collagen hydrogel which surrounds the aligned fibers comprises of therapeutics of interest, which could provide sustained delivery of biochemical signaling to further facilitate nerve regeneration.

In our previous work,^[^ ^16^ ^]^ we used SCI model as a proof‐of‐concept to demonstrate that miRs can enhance axon regeneration in vivo at 2 weeks post SCI. However, a limitation of that work is the lack of long time point studies, animal behavioral tests, as well as analyses of the mechanisms behind our observations. Therefore, in this work, we further demonstrate that scaffold‐mediated delivery of a cocktail of Axon miRs, along with glial cell‐derived neurotrophic factor (GDNF), greatly promoted and preserved matured axon regeneration and survival (ascending sensory axons and descending serotonergic axons) following a severe SCI in rodents at week 12 post SCI. Additionally, when coupled with methylprednisolone, a glucocorticoid steroid claimed previously to improve neurological recovery in SCI human patients,^[^ ^17^ ^]^ both sensory and motor recovery were further enhanced. In‐depth analysis of the regulated genes using RNA sequencing suggests that in the presence of methylprednisolone, Axon miRs treatment decreased pro‐inflammatory response and enhanced the expression of beneficial extracellular matrix (ECM)‐related genes, which may account for the observed anatomical and functional restoration.

## 2 Results

### 2.1 Fiber‐Hydrogel Scaffold for Sustained Release of Biomolecules was Successfully Fabricated

**Figure 1:**
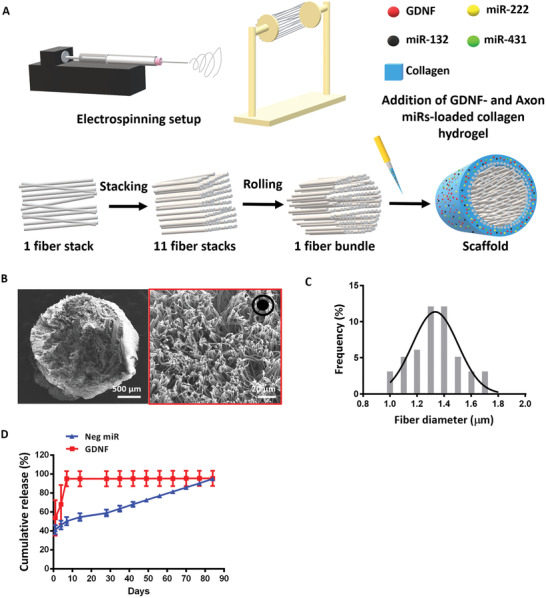
Fiber‐hydrogel scaffold was successfully fabricated. A) Scaffold fabrication schematic diagram. B,C) SEM image of the entire scaffold and high magnification of PCL electrospun fibers (red box). A total of 50 fibers were quantified and the average fiber diameter was 1.35 ± 0.19 µm. Black arrow pointing out of the paper at the top right‐hand corner of (B) indicates the directionality of the fibers. D) Cumulative release of Neg miR and GDNF over time. All data are represented as mean ± SD.

Prolonged availability of biomolecules is desirable for the treatment of SCI as the injured microenvironment is highly dynamic and requires biochemical guidance in order to achieve nerve regeneration and subsequent functional recovery. We first demonstrate that the fiber‐hydrogel scaffold used in this study is capable of imparting topographical (inner aligned fibers) (**Figure** **1**A–C) and biochemical cues (loaded miRs and neurotrophic factor) for regenerating injured tissues. Importantly, a sustained release of the encapsulated biomolecules was obtained over a period of 3 months for the miRs and 1 week for GDNF (Figure 1D). Besides that, the loading efficiency of Axon miRs and GDNF was 5.25% and 11.86%, respectively. The potential of GDNF in promoting neuronal survival, axon sprouting and functional recovery after SCI has been well documented.^[^ ^18^, ^19^, ^20^ ^]^ Besides that, as an important neurotrophic factor for the development of the nervous system, GDNF was also shown to reduce secondary damage and decrease lesion size after SCI.^[^ ^18^, ^21^ ^]^ To supplement this study, the effect of GDNF on spinally injured rats was evaluated at week 2 and we demonstrated that as compared to Untreated rats, GDNF‐treated rats exhibited significantly more robust axon regeneration (*p* < 0.01, Figure S1, Supporting Information).

### 2.2 Axon MicroRNAs Promoted Significant Regeneration of Mature Axons at Injury Site, as well as Serotonergic and Sensory Axons at Rostral and Caudal Regions of Injury Site Respectively

**Figure 2:**
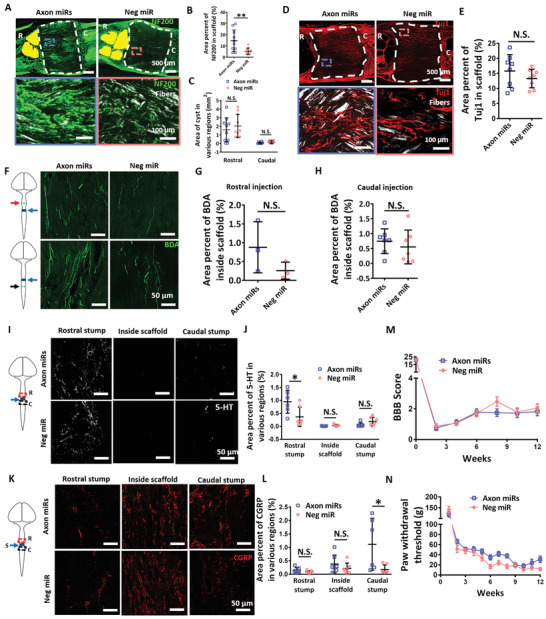
Axon miRs promoted significant regeneration of mature axons at injury site, as well as, serotonergic and sensory axons at rostral and caudal regions of injury site respectively. A) Representative fluorescent images of NF200 staining in Axon miRs‐ and Neg miR‐treated rats at 12 weeks post SCI. Images shown in the second row are the enlarged images of the boxed areas in the first row. B) Quantification analysis of area percent occupied by NF200^+^ signals within the scaffold region in Axon miRs‐ and Neg miR‐treated rats. Results indicate that treatment with Axon miRs enhanced nerve regeneration. **: *p* < 0.01, Student's *t*‐test. *N* = 10 in each group. C) Quantification analysis of cyst size (labelled as yellow) at both rostral and caudal of the injured spinal cord. Student's *t*‐test between Axon miRs and Neg miR group for both rostral and caudal region. *N* = 10 in each group. D,E) Representative fluorescent images of Tuj1 staining at 12 weeks post SCI. Quantification analysis of area percent occupied by Tuj1+ signals within the scaffold region. Student's *t*‐test. *N* = 8 in each group. F–H) Schematic diagram and representative fluorescent images of BDA^+^ signals inside the scaffold; Quantification analysis of area percent occupied by BDA^+^ signals, suggest that treatment with Axon miRs did not affect the regeneration of propriospinal axons within the spinal cord. Student's *t*‐test. *N* = 3 for each group in (G) and *N* = 7 for each group in (H). I,J) Representative fluorescent images of 5‐HT^+^ serotonergic axons in Axon miRs‐ and Neg miR‐treated rats at 12 weeks post SCI. Quantification analysis of area percent occupied by 5‐HT^+^ signals at rostral region, within the scaffold and caudal region, suggests that Axon miRs treatment promoted the regeneration of serotonergic axons at rostral region. *: *p* < 0.05, Student's *t*‐test between Axon miRs and Neg miR group for all regions. *N* = 6 in each group. K,L) Representative fluorescent images of CGRP staining in Axon miRs‐ and Neg miR‐treated rats at 12 weeks post SCI. Quantification analysis of area percent occupied by CGRP^+^ sensory axons at rostral region, within the scaffold and caudal region, suggests that Axon miRs treatment promoted the regeneration of ascending sensory axons at caudal region. *: *p* < 0.05, Student's *t*‐test between Axon miRs and Neg miR group for all regions. *N* = 6 in each group. M) BBB scores obtained at bi‐weekly intervals from Axon miRs‐ and Neg miR‐treated rats indicate that treatment with Axon miRs alone did not enhance motor recovery. Student's *t*‐test between Axon miRs and Neg miR group for each time point. *N* = 10 in each group. N) Von Frey Hair test, which was conducted by measuring the paw withdrawal threshold at weekly intervals in Axon miRs‐ and Neg miR‐treated rats. Results indicate that treatment with Axon miRs alone did not enhance sensory function recovery. Student's *t*‐test between Axon miRs and Neg miR group for each time point. *N* = 10 in each group. All data are represented as mean ± SD, except for (M) and (N) which are shown in mean ± SEM.

We previously showcased the potency of Axon miRs in enhancing axonal regeneration at an early time point (2 weeks).^[^ ^16^ ^]^ Here, the effect of Axon miRs was reproduced even when the recovery process was prolonged for a longer time point of 12 weeks (14.70 ± 9.478% vs 5.252 ± 2.910%, *p* < 0.01) (**Figure** **2**A,B) and this occurred despite cyst formation in the rostral stump of the transected spinal cord (Figure 2C). Additionally, we found that, through direct quantification, Axon miRs treatment enhanced the formation of NF200^+^ mature axons but not Tuj‐1^+^ immature axons (Figure 2D,E). Biotinylated dextran amine (BDA)‐labelled propriospinal axons were found to be unaffected by Axon miRs treatment as well (Figure 2F,H). Notably, the descending 5‐HT^+^ serotonergic projections (0.949 ± 0.446% vs 0.367 ± 0.370%, *p* < 0.05, Figure 2I,J), as well as, the ascending CGRP^+^ primary afferents from dorsal root ganglion cells^[^ ^22^ ^]^ (1.113 ± 0.969% vs 0.178 ± 0.167%, *p* < 0.05, Figure 2K,L) were significantly preserved at the rostral and caudal sides of the scaffolds respectively after Axon miRs treatment. However, there was no notable enhancement in motor and sensory function recovery in the rats treated with Axon miRs throughout the experiments (Figure 2M,N). Additionally, treatment with Axon miRs did not lead to prominent glial scarring as compared to the Neg miR‐treated rats (Figure S3A,B, Supporting Information).

### 2.3 Administration of Axon MicroRNAs in the Presence of Methylprednisolone Synergistically Enhanced Function Recovery without Alteration in Axon Regeneration

**Figure 3:**
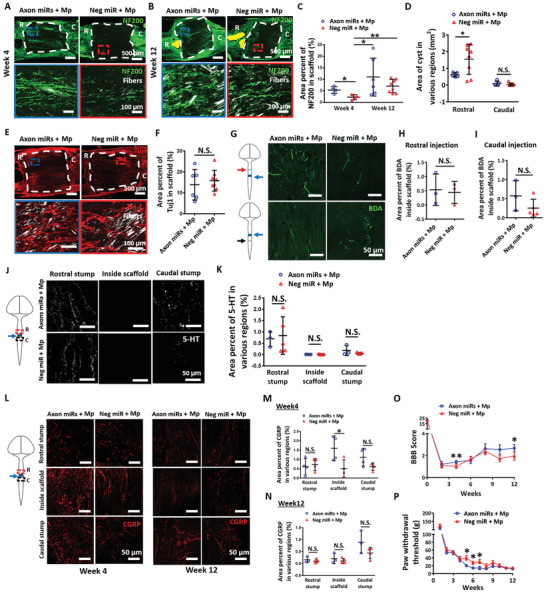
Administration of Axon miRs in the presence of methylprednisolone synergistically enhanced functional recovery without alteration in axon regeneration. A,B) Representative fluorescent images of NF200 staining in Axon miRs + Mp‐ and Neg miR + Mp‐treated rats at 4 and 12 weeks post SCI. Images shown in the second row are the enlarged images of the boxed areas from the first row. C) Quantification analysis of area percent occupied by NF200^+^ signals within the scaffold region in Axon miRs + Mp‐ and Neg miR + Mp‐treated rats at weeks 4 and 12. Results indicate that in the presence of methylprednisolone, Axon miRs treatment significantly enhanced nerve regeneration versus Neg miR. *p < 0.05; **p < 0.01, Shapiro‐Wilk normality test followed by Kruskal–Wallis test and Mann–Whitney post hoc test. These results, however, are not significantly different versus corresponding samples without methylprednisolone (Figure 2B). *N* = 4 in Axon miRs + Mp and Neg miR + Mp groups at week 4; *N* = 6 in Axon miRs + Mp group; and *N* = 8 in Neg miR + Mp group at week 12. D) Quantification analysis of cyst size (labelled as yellow) at rostral and caudal regions of the injury site at week 12, suggesting that in the presence of methylprednisolone, Axon miRs treatment decreased cavity size at rostral region versus Neg miR. *: *p* < 0.05, Student's *t*‐test between Axon miRs + Mp and Neg miR + Mp group for all regions. These results, however, are not significantly different versus corresponding samples without methylprednisolone (Figure 2C). *N* = 4 in each group at week 4; *N* = 6 in Axon miRs + Mp group; and *N* = 8 in Neg miR + Mp group at week 12. E,F) Representative fluorescent images of Tuj1 staining at 12 weeks post SCI. Quantification analysis of area percent occupied by Tuj1^+^ signals within the scaffold region, indicating that Axon miRs did not affect immature neurons even in the presence of methylprednisolone. Student's *t*‐test. These results are also not significantly different versus corresponding samples without methylprednisolone (Figure 2E). *N* = 6 in Axon miRs + Mp group and *N* = 8 in Neg miR + Mp group. G–I) Schematic diagram and representative fluorescent images of BDA^+^ signals inside the scaffold. Quantification analysis of area percent occupied by BDA^+^ signals once again suggests that Axon miRs did not significantly enhance the regeneration of propriospinal axons even in the presence of methylprednisolone. Student's *t*‐test. These results are also not significantly different versus corresponding samples without methylprednisolone (Figure 2G,H). *N* = 3 in each group for (G); *N* = 3 in Axon miRs + Mp group; and *N* = 5 in Neg miR + Mp group for (H). J,K) Representative fluorescent images of 5‐HT staining in Axon miRs + Mp‐ and Neg miR + Mp‐treated rats at 12 weeks post SCI. Quantification analysis of area percent occupied by 5‐HT^+^ serotonergic axons at rostral region, within the scaffold and caudal region. Results suggest that in the presence of methylprednisolone, Axon miRs did not enhance the regeneration of 5‐HT^+^ serotonergic axons versus Neg miR. Student's *t*‐test between Axon miRs + Mp and Neg miR + Mp group for all regions. These results are also not significantly different versus corresponding samples without methylprednisolone (Figure 2J). *N* = 3 in Axon miRs + Mp group and *N* = 5 in Neg miR + Mp group. L–N) Representative fluorescent images of CGRP staining in Axon miRs + Mp‐ and Neg miR + Mp‐treated rats at 4 and 12 weeks post SCI. Quantification analysis of area percent occupied by CGRP+ sensory axons at rostral region, within the scaffold and caudal region. Results suggest that in the presence of methylprednisolone, as compared to Neg miR treatment, Axon miRs enhanced the regeneration of CGRP^+^ sensory axons within the scaffold at week 4, but had no significant effect at week 12. *p < 0.05, Student's *t*‐test between Axon miRs + Mp and Neg miR + Mp group for all regions. These results are also not significantly different versus corresponding samples without methylprednisolone (Figure 2L). *N* = 3 in Axon miRs + Mp group and *N* = 5 in Neg miR + Mp group. O) BBB scores obtained at bi‐weekly intervals from weeks 1 to 12 indicate that in the presence of methylprednisolone, Axon miR treatment enhanced motor recovery at week 12. *p < 0.05; **p < 0.01, Student's *t*‐test between Axon miRs + Mp and Neg miR + Mp group for each time point. *N* = 6 in Axon miRs + Mp group and *N* = 8 in Neg miR + Mp group. P) Von Frey Hair test, which was indicated by the paw withdrawal threshold as measured at weekly intervals from weeks 1 to 12, indicate that in the presence of methylprednisolone, Axon miRs enhanced rate of sensory function recovery. *p < 0.05, Student's *t*‐test between Axon miRs and Neg miR group for each time point. *N* = 6 in Axon miRs + Mp group and *N* = 8 in Neg miR + Mp group. All data were represented as mean ± SD, except for (O) and (P) which are shown in mean ± SEM.

In an attempt to achieve both axonal regeneration, as well as, functional recovery, we next administered methylprednisolone, an anti‐inflammatory steroid and a possible neuroprotectant, along with the Axon miRs and assessed the outcomes at both weeks 4 and 12. Correspondingly, in the presence of methylprednisolone, we observed notable improvements in mature axon regeneration in Axon miRs‐treated rats for both week 4 (i.e., 5.375 ± 1.455% vs 2.254 ± 1.095%, *p* < 0.05) and week 12 (11 ± 8.219% vs 7.079 ± 2.889%) (**Figure** **3**A–C), as compared to Neg miR‐treated rats. These results, however, are not significantly different in contrast to corresponding samples without methylprednisolone (Figure 2B and Figure S4, Supporting Information). Besides that, in the presence of methylprednisolone, treatment with Axon miRs also led to a significant reduction of the cyst size in the rostral stump of the injured cord (*p* < 0.05) (Figure 3D). These results, however, are not significantly different versus corresponding samples without methylprednisolone (Figure 2C and Figure S5, Supporting Information). Additionally, the immature axons (Figure 3E,F), the propriospinal axons (Figure 3G–I), as well as, the 5‐HT^+^ serotonergic axons (Figure 3J,K) appeared to be unaffected, indicating that Axon miRs did not affect these subgroups of neurons even in the presence of methylprednisolone. These results are also not significantly different as compared to corresponding samples without methylprednisolone (Figure 2D–J and Figures S6 to S8, Supporting Information).

On the other hand, results suggest that in the presence of methylprednisolone, Axon miRs enhanced the regeneration of CGRP^+^ sensory axons within the scaffold at week 4 (*p* < 0.05) but had no significant effects at week 12 (Figure 3L–N). These results are also not significantly different in contrast to corresponding samples without methylprednisolone (Figure 2L and Figure S9, Supporting Information). More importantly, administration of Axon miRs in the presence of methylprednisolone displayed marked motor and sensory function recovery at week 12 (*p* < 0.05) (Figure 3O) and week 5 to 7 (*p* < 0.05) (Figure 3P) respectively, suggesting that Axon miRs enhanced motor function and the rate of sensory function recovery in the presence of methylprednisolone. Of note, we observed that Axon miRs treatment led to significant glial scarring as compared to Neg miR‐treated rats in the presence of methylprednisolone at week 12 (61.11 ± 10.24% vs 40.22 ± 16.86%, *p* < 0.05, Figure S3C,D, Supporting Information). Besides that, in the presence of methylprednisolone, the glial scarring exhibited in Axon miRs‐treated rats at week 12 is also significantly more as compared to its corresponding earlier time point at week 4 (61.11 ± 10.24% vs 28.07 ± 5.683%, *p* < 0.05) (Figure S3C,D, Supporting Information). However, results at week 12 were also not significantly different versus corresponding samples without methylprednisolone (Figures S3B and S10, Supporting Information).

### 2.4 Spinal Cord Transection Led to Massive Gene Dysregulation 1 Week after Injury

**Figure 4:**
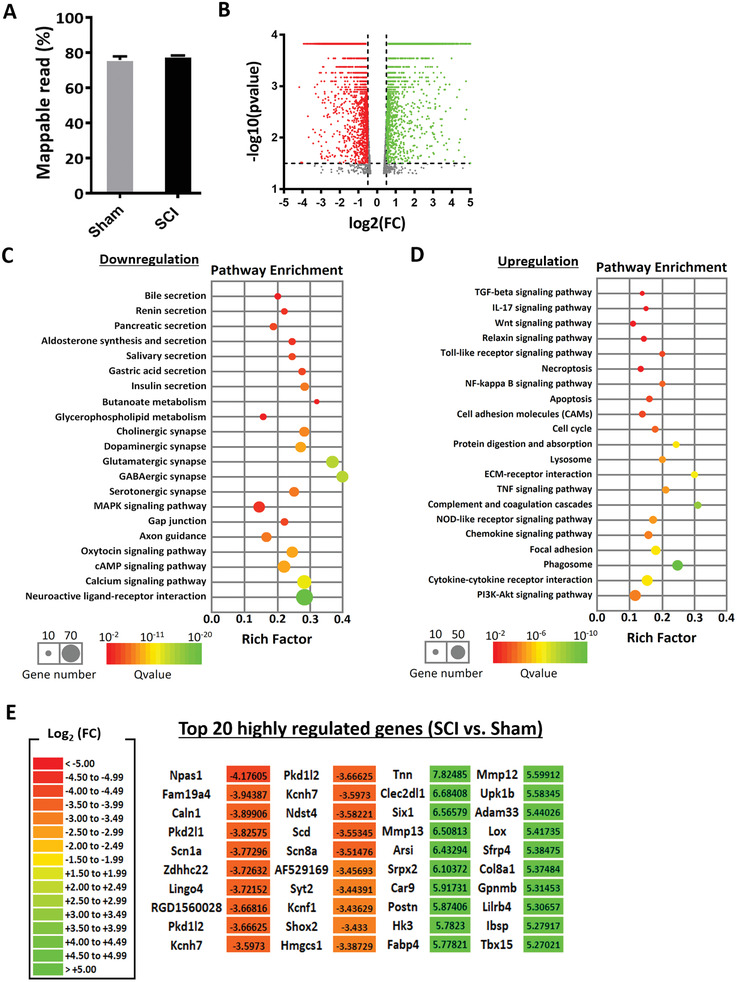
Spinal cord transection led to massive gene dysregulation 1 week after injury. A) The mappable reads in Sham (*n* = 3) and SCI (*n* = 2) rats are similar. B) Volcano plot of DEGs in SCI as compared to Sham. Red and green dots represent the down‐ and upregulated genes after SCI, respectively, whereas grey dots show genes with no significant changes. Significant DEGs have fold change of ±1.5, and *p*‐value ≤ 0.05. C,D) KEGG pathway enriched in the downregulated and upregulated genes in SCI compared to Sham, respectively. E) Top 20 significantly regulated genes. Red and green colours represent the most significantly downregulated and upregulated genes, respectively.

The transcriptome profile of a spinal cord transacted rat has not been well‐studied, and studying the gene expression profile changes will allow us to better understand the progress of spinal cord transection injury and explore potential therapeutics. Here, we demonstrate that for a fully transacted spinal cord, on the conditions of quality‐harvested RNAs (Figure S11, Supporting Information) and sufficient mappable reads (**Figure** **4**A), we detected thousands of significantly (*q* < 0.05) dysregulated genes in total in SCI samples as compared to Sham uninjured samples (Figure 4B). Genes that were downregulated affected notable processes like secretion (pancreatic, gastric acid, insulin, etc.) and synapses (cholinergic, dopaminergic, glutamatergic, etc.) (Figure 4C). Conversely, upregulated genes mostly corresponded to immune‐related signaling pathways (IL‐17, Toll‐like receptor, TNF, chemokine, etc.), as well as, cell death (apoptosis and necroptosis) (Figure 4D). A complete list of the up‐ or downregulated genes can be found from the excel file in Supporting Information, namely SCI versus Sham_1.5 fold in Supporting Information. For conciseness, we also included a list of the top 20 highly regulated genes which underwent the largest fold change (Figure 4E).

### 2.5 Treatment with Axon MicroRNAs in the Presence of Methylprednisolone Decreased the Expression of Pro‐Inflammatory Related Genes and Enhanced Genes Related to Extracellular Matrix Deposition

**Figure 5:**
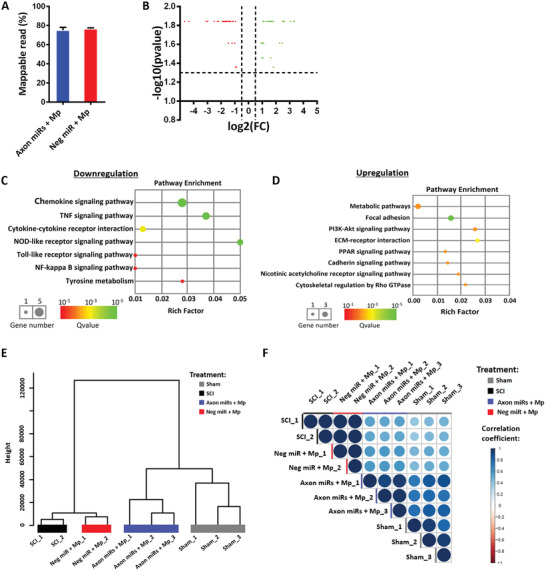
Treatment with Axon miRs in the presence of methylprednisolone decreased the expression of pro‐inflammatory related genes and enhanced genes related to ECM deposition. A) The mappable reads in Axon miRs + Mp‐ (*n* = 3) and Neg miR + Mp‐treated (*n* = 2) groups are similar. B) Volcano plot of DEGs in Axon miRs + Mp‐ and Neg miR + Mp‐treated groups. Red dots represent the downregulated genes, and green dots represent the upregulated genes in Axon miRs + Mp‐treated samples versus Neg miR + Mp‐treated samples. Significant DEGs have fold change of ±1.5, and *p*‐value ≤ 0.05. C,D) KEGG pathway enrichment of the downregulated and upregulated genes, respectively. The rich factor was calculated by the number of significantly regulated genes in a certain pathway divided by the number of all background genes in that pathway. E) Hierarchical clustering of samples based on their overall gene expression profiles. Segregation of Axon miRs + Mp‐treated samples with Sham samples, and Neg miR + Mp‐treated samples with SCI samples were observed. F) Transcriptome correlation plot of all samples, where the extent of positive correlation between replicates is indicated by the extent of dark blue shading and large circle size. Results indicate good correlation between Axon miRs + Mp‐treated rats and Sham samples.

We next compared the genes that were altered when the Axon miRs were utilized against Neg miR in the presence of methylprednisolone so as to reveal the possible mechanisms behind this treatment. In our previous work, the Axon miRs were demonstrated to downregulate their downstream targets, such as PTEN, Rasa1, and Kremen1, in primary rat cortical neurons, as well as, lengthen their neurite outgrowth in vitro.^[^ ^16^ ^]^ However, when administered in vivo, and analyzed under stringent RNA quality control (Figure S11, Supporting Information, and **Figure** **5**A), none of the 62 genes that were significantly dysregulated (*q* < 0.05) (Figure 5B) in Axon miRs as compared to Neg miR were the known downstream targets of Axon miRs. Accordingly, the downregulated genes were mostly involved in eliciting immune responses (Figure 5C) while the upregulated genes were associative with metabolic and ECM pathways (Figure 5D). Additionally, treatment of Axon miRs in the presence of methylprednisolone resulted in lower numbers of iNOS^+^Iba1^+^ microglial within the scaffold and at the implant interface (Figure S12, Supporting Information).

Interestingly, hierarchical clustering based on the overall gene expression profiles indicated that Axon miRs + Mp‐treated samples exhibited more similar expression profile to Sham samples, while Neg miR + Mp‐treated samples had more similar expression profiles to SCI samples (Figure 5E). Furthermore, transcription correlation analysis of all samples, where the extent of positive correlation between replicates is indicated by the extent of dark blue shading and large circle size, indicated good correlation between Axon miRs + Mp‐treated rats and Sham samples (*r* ^2^ ranges from 0.717 to 0.878 between Axon miRs + Mp‐treated rats and Sham replicates, Figure 5F). For a clearer observation, we further examined the 62 genes that are significantly regulated in Axon miRs + Mp‐treated samples in contrast to the Neg miR + Mp‐treated samples and plotted a heatmap based on their gene expression in all four groups in Figure S12, Supporting Information. This allowed us to identify a subgroup of ECM related genes (e.g., Lyve1, Dmp1, and Ctsk) that were upregulated only in Axon miRs + Mp‐treated rats but showed little change in expression in response to SCI alone or Neg miR + Mp treatment.

### 2.6 Axon MicroRNAs Treatment Alone Enhanced Myelination and Synapse Formation But may Have Less Impact in the Presence of Methylprednisolone

**Figure 6:**
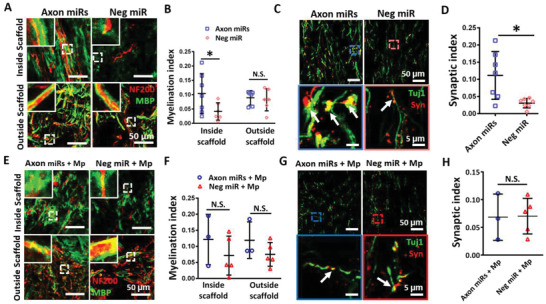
Axon miRs treatment alone enhanced myelination and synapse formation but may have less impact in the presence of methylprednisolone. A) Representative fluorescent images of NF200 and MBP staining in Axon miRs‐ and Neg miR‐treated rats. The insets at top left corners show the enlarged images of the boxed area from the low magnification images. B) Quantification analysis of myelination index at both inside the scaffold and the tissue‐scaffold interface (i.e., both rostral and caudal). Results suggest that treatment with Axon miRs significantly promoted myelination inside the scaffold. *: *p* < 0.05, Student's *t*‐test between Axon miRs and Neg miR group for all regions. *N* = 7 in Axon miRs group and *N* = 6 in Neg miR group. C) Representative fluorescent images of Tuj1 and synaptophysin staining in Axon miRs‐ and Neg miR‐treated rats. Images in the second row show the enlarged images of the boxed region in the low magnification images in the first row. D) Quantification analysis of synaptic index inside the scaffold in both Axon miRs‐ and Neg miR‐treated rats. Data suggest that treatment of Axon miRs significantly promoted synapse formation. *: *p* < 0.05, Student's *t*‐test between Axon miRs and Neg miR group. *N* = 7 in each group. E) Representative fluorescent images of NF200 and MBP staining in Axon miRs + Mp‐ and Neg miR + Mp‐treated rats. The insets at top left corners show the enlarged images of the boxed area from the low magnification images. F) Quantification analysis of myelination index at both inside the scaffold and the tissue‐scaffold interface (i.e., both rostral and caudal). Data indicate that in the presence of methylprednisolone, treatment with Axon miRs did not enhance myelination both inside and outside the scaffold versus Neg miR treatment. Student's *t*‐test between Axon miRs + Mp and Neg miR + Mp group for all regions. These results are also not significantly different when compared with corresponding samples without methylprednisolone (Figure 6B). *N* = 3 in Axon miRs + Mp group and *N* = 5 in Neg miR + Mp group. G) Representative fluorescent images of Tuj1 and synaptophysin staining in Axon miRs + Mp‐ and Neg miR + Mp‐treated rats. Images in the second row show the enlarged images of the boxed region in the low magnification images in the first row. H) Quantification analysis of synaptic index inside the scaffold. Data indicate that in the presence of methylprednisolone, treatment of Axon miRs did not enhance synapse formation inside the scaffold versus Neg miR treatment. Student's *t*‐test between Axon miRs + Mp and Neg miR + Mp group. These results are also not significantly different when compared with corresponding samples without methylprednisolone (Figure 6F). *N* = 3 in Axon miRs + Mp group and *N* = 5 in Neg miR + Mp group. All data were represented as mean ± SD.

Having elucidated the possible mechanisms that contributed to the recovery after SCI, we further explored whether the structures that confer axon functionality, namely, myelin sheath and synaptic connections, were affected by these biomolecules as well. We noticed that at week 12, when Axon miRs were administered alone, it significantly enhanced the myelination index inside the scaffolds (0.104 ± 0.070% vs 0.042 ± 0.030%, *p* < 0.05; **Figure** **6**A,B), as well as, the synaptic index within the scaffolds (0.112 ± 0.070% vs 0.031 ± 0.014%, *p* < 0.05; Figure 6C,D), as compared to Neg miR control. However, in the presence of methylprednisolone, Axon miRs treatment only showed a trend of enhanced myelination inside and outside the scaffolds but the results were not significant versus Neg miR + Mp treatment (Figure 6E,F). These results, were also not significantly different when compared with corresponding samples without methylprednisolone (Figure 6B and Figure S14, Supporting Information). Additionally, in the presence of methylprednisolone, Axon miRs treatment also did not appear to facilitate synapse formation within the scaffolds (Figure 6G,H). These results are also not significantly different when compared with corresponding samples without methylprednisolone (Figure 6D and Figure S15, Supporting Information). While methylprednisolone may have some effects on Axon miRs treatment, it is also possible that the small sample sizes in methylprednisolone treated animals may have resulted in the lack of statistical significance. Specifically, part of the samples (*n* = 3 from each group) were subjected to both anterograde and retrograde labelling. To differentiate them, normal BDA was injected caudally to the injured area and Texas Red tagged BDA was injected rostrally to the injured area. However, we realized that the fluorescence of Texas Red BDA occupied both Cy3 and Cy5 channels. As a result, there were less animals that could be used for quantification in Figure 6F,H, where double antibody staining was required. Taken together, these results suggest that Axon miRs treatment alone enhanced myelination and synapse formation but the impact appeared diminished in the presence of methylprednisolone.

### 2.7 Increased Axon MicroRNAs Dosage (Boosted Axon MicroRNAs) in the Presence of Methylprednisolone Significantly Promoted Functional Recovery and Myelination Index but did not Alter Axon Regeneration

**Figure 7:**
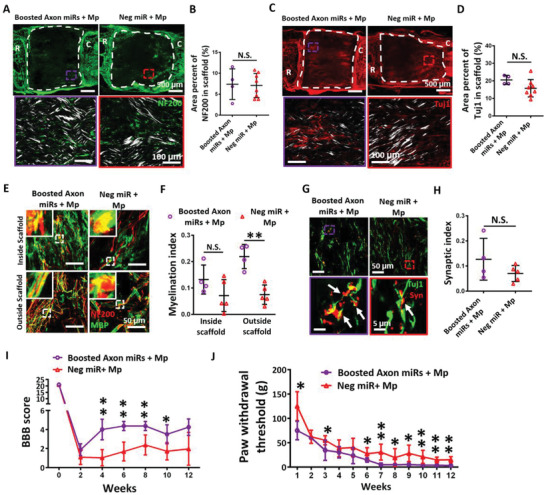
Increased Axon miRs dosage (Boosted Axon miRs) in the presence of methylprednisolone significantly promoted functional recovery and myelination index but did not alter axon regeneration. A) Representative fluorescent images of NF200 staining in Boosted Axon miRs + Mp‐ and Neg miR + Mp‐treated groups. Images in the second row show the enlarged images of the boxed region in the low magnification images in the first row. B) Quantification analysis of area percent occupied by NF200^+^ signals within the scaffolds. Results suggest that treatment of Boosted Axon miRs + Mp did not significantly enhance nerve regeneration. Student's *t*‐test between Boosted Axon miRs + Mp and Neg miR + Mp group. These results are also not significantly different when compared with corresponding samples with lower Axon miR dosage (Figure 3C) and without methylprednisolone (Figure 2B). *N* = 4 in boosted Axon miRs + Mp group and *N* = 8 in Neg miR + Mp group. C) Representative fluorescent images of Tuj1 staining in Boosted Axon miRs + Mp‐ and Neg miR + Mp‐treated rats. Images in the second row show the enlarged images of the boxed region in the low magnification images in the first row. D) Quantification analysis of area percent occupied by Tuj1^+^ signals within the scaffold in Boosted Axon miRs + Mp‐treated rats versus Neg miR + Mp‐treated rats (*p* = 0.089). Student's *t*‐test between Boosted Axon miRs + Mp and Neg miR + Mp group. These results are not significantly different when compared with corresponding samples with lower Axon miR dosage (Figure 3F) and without methylprednisolone (Figure 2E). *N* = 4 in boosted Axon miRs + Mp group and *N* = 8 in Neg miR + Mp group. E) Representative fluorescent images of NF200 and MBP staining in Boosted Axon miRs + Mp‐treated animals and Neg miR + Mp‐treated animals. The insets at top left corner show the enlarged images of the boxed area in the low magnification images. F) Quantification analysis of myelination index at both inside the scaffold and the tissue‐scaffold interface in Boosted Axon miRs + Mp‐ and Neg miR + Mp‐treated rats. Results suggest that treatment with Boosted Axon miRs + Mp promoted myelination. **: *p* < 0.01, Student's *t*‐test between Boosted Axon miRs + Mp and Neg miR + Mp group for all regions. The myelination index outside the scaffolds as obtained under high dosage of Axon miRs is also significantly higher as compared to rats treated with lower Axon miR dosage (vs Figure 6D, outside scaffold, *p* = 0.077) and in the absence of methylprednisolone (vs Figure 6F, outside scaffold, *p* < 0.011). *N* = 4 in boosted Axon miRs + Mp group and *N* = 5 in Neg miR + Mp group. G) Representative fluorescent images of Tuj1 and synaptophysin staining in Boosted Axon miRs + Mp‐ and Neg miR + Mp‐treated rats. Images in the second row show the enlarged images of the boxed region in the low magnification images in the first row. H) Quantification analysis of synaptic index inside the scaffolds. Student's *t*‐test between Boosted Axon miRs + Mp and Neg miR + Mp group for all regions. These results are not significantly different when compared with corresponding samples with lower Axon miR dosage (Figure 6H) and without methylprednisolone (Figure 6F). *N* = 4 in boosted Axon miRs + Mp group and *N* = 5 in Neg miR + Mp group. I) BBB scores as obtained at bi‐weekly intervals from Boosted Axon miRs + Mp‐ and Neg miR + Mp‐treated rats from weeks 1 to 12. *: *p* < 0.05; **: *p* < 0.01, Student's *t*‐test between Boosted Axon miRs + Mp and Neg miR + Mp group for each time point. *N* = 4 in boosted Axon miRs + Mp group and *N* = 8 in Neg miR + Mp group. J) Von Frey Hair test, which is indicated by the paw withdrawal threshold in Boosted Axon miRs + Mp‐ and Neg miR + Mp‐treated rats as assessed at weekly intervals from weeks 1 to 12. Functional assessments suggest that the increased Axon miRs dosage significantly promoted the rate of sensory function recovery and the extents of motor and sensory function recovery. Student's *t*‐test between Boosted Axon miRs + Mp and Neg miR + Mp group for each time point. *N* = 4 in boosted Axon miRs + Mp group and *N* = 8 in Neg miR + Mp group. All data were represented as mean ± SD, except for (I) and (J) which are shown in mean ± SEM.

Next, we wondered if the functional recovery of the injured rats can be further enhanced by increasing the amount of Axon miRs administered. To test this idea, we conducted another experiment where rats received twice the dosage of Axon miRs. We observed that in the presence of methylprednisolone, Boosted Axon miRs did not enhance NF200^+^ mature axon regeneration (**Figure** **7**A,B), as compared to Neg miR control. These results were not significantly different when compared with corresponding samples with lower Axon miR dosage (Figure 3C and Figure S4, Supporting Information) and without methylprednisolone (Figure 2B and Figure S4, Supporting Information). Besides that, in the presence of methylprednisolone, the Tuj‐1^+^ immature axons were not significantly enhanced by the treatment of Boosted Axon miRs (Figure 7C,D) as well. These results were also not significantly different when compared with corresponding samples with lower Axon miR dosage (Figure 3F and Figure S6, Supporting Information) and without methylprednisolone (Figure 2E and Figure S6, Supporting Information).

In order to know how the functional structures were affected by Boosted Axon miRs, myelination, and synapse formation were then investigated in Boosted Axon miRs‐treated rats. Correspondingly, we noted that in the presence of methylprednisolone, treatment with Boosted Axon miRs promoted myelination, especially outside the scaffolds (0.220 ± 0.046% vs 0.075 ± 0.037%, *p* < 0.05; Figure 7E,F). The myelination index outside the scaffolds as obtained under high dosage of Axon miRs was also significantly higher as compared to rats treated with lower Axon miR dosage (vs Figure 6F, outside scaffold, *p* = 0.077, Figure S14, Supporting Information) and in the absence of methylprednisolone (vs Figure 6B, outside scaffold, *p* < 0.05, Figure S14, Supporting Information). Additionally, in the presence of methylprednisolone, treatment with Boosted Axon miRs did not enhance the synaptic index (Figure 7G,H). These results were also not significantly different when compared with corresponding samples with lower Axon miR dosage (Figure 6H and Figure S15, Supporting Information) and without methylprednisolone (Figure 6F and Figure S15, Supporting Information).

Importantly, in the presence of methylprednisolone, the rats treated with Boosted Axon miRs exhibited relatively exceptional motor (Figure 7I, *p* < 0.01) and sensory (Figure 7J, *p* < 0.01) recovery, as compared to Neg miR‐treated rats. These functional assessments suggest that the increased Axon miRs dosage significantly promoted the rate of sensory function recovery and the extents of motor and sensory function recovery. Taken together, these results confirmed that treatment with Axon miRs in the presence of methylprednisolone significantly promoted functional recovery and myelination index without altering axon regeneration. Finally, we noted that glial scar formation in Boosted Axon miRs‐treated rats was significant lower when compared to its corresponding samples with lower Axon miR dosage (26.98 ± 4.39 vs 61.11 ± 10.24, *p* < 0.05, Figures S3D and S10, Supporting Information) and without methylprednisolone (26.98 ± 4.39 vs 57.04 ± 27.83, *p* = 0.089, Figures S3B and S10, Supporting Information).

## 3 Discussion

A major cause of regeneration failure after SCI is the diminished regenerative ability of mature CNS neurons.^[^ ^23^, ^24^ ^]^ As such, while the conventional method of providing a conducive environment for regenerating neurons is important, it is also essential to stimulate the intrinsic growth ability of neurons. Here, we engineered a 3D fiber‐hydrogel scaffold which can be directly implanted into the transected rat spinal cord to provide a supporting environment that directs the regrowth of axons through aligned fiber contact guiding signals and GDNF, as well as, sustained and localized availability of miRs^[^ ^25^ ^]^ that are known to enhance axon intrinsic growth ability.^[^ ^6^, ^11^, ^12^, ^13^, ^15^, ^26^ ^]^

As indicated in our RNA‐sequencing analysis (Figure 4), a fully transected rat spinal cord has a highly dysregulated transcriptome with thousands of genes significantly dysregulated at 7 days post injury. Many of these dysregulated genes were very similar to that seen in a rat contusion model (1421 dysregulated genes) by Li et al.^[^ ^27^ ^]^ Some of the highly upregulated genes included plasminogen activator, urokinase receptor (Plaur), complement component 5a receptor 1 (C5ar1), chemokine ligand 2 (Ccl2), CD68, chemokine ligand 6 (Ccl6), serpin peptidase inhibitor, clade E member 1 (Serpine1), plasminogen activator (Plau), macrophage scavenger receptor 1 (Msr1), heme oxygenase (decycling) 1 (Hmox1), and suppressor of cytokine signaling 3 (Socs3). Unfortunately, we did not observe similar downregulated genes as Li and colleagues.^[^ ^27^ ^]^ Surprisingly, the genes dysregulated in mice with a fully transected spinal cord^[^ ^28^ ^]^ differed from our results more than the spinal cord of contused rats, despite analyzing similar time points. For example, notable genes related to the oligodendroglial cells and myelin, such as, oligodendrocyte transcription factor 1 (Olig1), oligodendrocyte transcription factor 2 (Olig2), SRY‐box transcription factor 6 (Sox6), 2’,3’‐cyclic nucleotide 3’ phosphodiesterase (Cnp), transmembrane protein 63a (Tmem63a), and proteolipid protein 1 (Plp1) were upregulated in mice but downregulated in our samples. Similar trends were observed for other genes, such as, SRY‐box transcription factor 2 (Sox2) and SRY‐box transcription factor 10 (Sox10, involved in embryonic development), calbindin 2 (Calb2), and hepatic and glial cell adhesion molecule (Hepacam).^[^ ^28^ ^]^ Hence, these comparisons suggest that the genes that are dysregulated following SCI may be more dependent on the species than on the type of injury. This notion has huge implications and more work should be conducted for validation.

Given that a plethora of genes were dysregulated after SCI, it may be essential to modulate several genes at the same time in order to elicit tissue regrowth and function recovery. In this respect, miRs are ideal therapeutic molecules, since miRs can regulate the expression of several genes concurrently. In addition, using miR therapeutics might be beneficial over the more commonly‐adopted growth factor therapy, as most growth factors offer selective support for only the subclass of neurons that express the appropriate receptors to allow neurons to take up these factors. In cases where the expression levels of the receptors are low and nerve damage involves a wide spectrum of neurons, such as, in SCI, growth factor‐based approaches may be less effective.^[^ ^4^ ^]^

Hence, Axon miRs (miR‐132,^[^ ^6^, ^11^, ^15^, ^26^ ^]^ miR‐222^12^, and miR‐431^13^) were chosen as the therapeutic molecules in this work. These miRs are enriched in axons^[^ ^9^, ^15^, ^29^ ^]^ and are significantly altered after nerve injuries.^[^ ^12^, ^13^, ^30^, ^31^, ^32^, ^33^ ^]^ Importantly, in our previous work, through an exhaustive screening process, we identified this combination of miRs to be optimal in enhancing nerve regeneration after SCI.^[^ ^16^ ^]^ In addition, these Axon miRs effectively promoted neurite outgrowth from neurons in vitro, regardless of the age (embryonic, post‐natal, adult) and origin (CNS, PNS)^[^ ^16^ ^]^ of the neurons. Therefore, we believe this Axon miRs treatment should be widely applicable to patients of different age who suffer from acute nerve injuries.

Mechanistically, when the expression of miR‐132, miR‐222, and miR‐431 were individually increased in the injured adult neurons, enhanced regeneration occurred from the growth cones.^[^ ^12^, ^13^, ^33^ ^]^ These miRs either enhance pathways that regulate neurogenesis and axon growth (e.g., miR‐431 enhances Wnt signaling which is needed for neurogenesis and axon growth by decreasing the expression of Kremen1, a Wnt antagonist^[^ ^13^ ^]^) or directly remove molecular brakes that prevent regrowth. These include PTEN (i.e., an inhibitor of the PI3K pathway that is important to central axon growth, for miR‐222^12^) and Ras GTPase, p120RasGAP (Rasa1) (i.e., involved in cytoskeletal regulation, for miR‐132^15^). In addition, miR‐222^12^ treatment also provided more significant increase in neurite outgrowth than PTEN^[^ ^4^, ^34^ ^]^ or Retinoblastoma^[^ ^35^ ^]^ siRNA treatment (≈2–3 times vs ≈1.5 times). These observations suggest the potency of controlling multiple gene targets by miR treatment as opposed to single mRNA regulation by siRNA.

Axon miRs (i.e., a cocktail of miR‐132, miR‐222, miR‐431) enhanced nerve regeneration at an early 2 week time point^16^ post SCI. Here, such improvements were also sustained over a longer time point of up to 12 weeks. Additionally, in our best animals, the density of regenerating mature axons that infiltrated the scaffold was nearly comparable to the density of the neural stem cells’ axonal projections from the scaffold into the host tissue in the landmark cell‐based study by Lu and Tuszynski et al.^[^ ^36^ ^]^

In the current work, methylprednisolone was administered to decrease the inflammatory reaction since the miRs utilized targeted nerve regeneration but not immune response. Within the drug dosages used in this study, Axon miRs significantly promoted axon regeneration (Figure 2B and Figure S4, Supporting Information), which remained unaffected by further inclusion of methylprednisolone treatment (Figure 3C and Figure S4, Supporting Information) and also by the increase in Axon miR dosage (Figure 7A and Figure S4, Supporting Information). Even though it seems obvious that adding both miRs and methylprednisolone will theoretically provide beneficial effects from both drugs, we demonstrated that relative therapeutics dosage is also crucial to achieve the desired outcomes. Specifically, when 5 µg of miRs were used in tandem with methylprednisolone, there was no obvious functional recovery. However, when 10 µg of miRs were utilized with methylprednisolone, the functional recovery of the injured rats was significantly enhanced (Figure 3O,P and Figure 7I,J). These outcomes suggest that there may be other factors, besides axon/neuron structures at the site of injury, that may lead to functional improvements. It is possible that the changes in glial cell involvement as associated with methylprednisolone and enhanced Axon miR dosage treatments, may have played significant roles toward functional improvements. Such can be seen by the increase in myelination index with increased Axon miR dosage (Figure 7F, outside scaffold), and the trend of decrease in iNOS^+^ pro‐inflammatory cells (Figure S12, Supporting Information), as well as, cyst formation (Figure 3D) in the presence of methylprednisolone. Additionally, Axon miRs treatment in the presence of methylprednisolone enhanced CGRP^+^ axons at 4 weeks post SCI (Figure 3M). Besides labelling sensory axons, CGRP also reflects the extent of immunoregulation occurring within the vicinity.^[^ ^37^ ^]^ A more crucial function of CGRP include a general negative regulation of inflammatory responses.^[^ ^38^, ^39^, ^40^ ^]^ Hence, as a result of the synergistic effects of Axon miRs and methylprednisolone, enhanced CGRP expression might be associative with the downregulation of various pro‐inflammatory pathways (Figure 5C,D) and the improved sensory function during the same time period (Figure 3P).

Similar to our tissue morphometric studies, bulk RNA‐seq analyses revealed the significant downregulation of the pro‐inflammatory genes (e.g., Ptx3, CCL7, CD74, and CXCL1) when animals were treated with Axon miRs + Mp. Additionally, genes related to ECM deposition (e.g., HAPLN1, Col2a1, and Ctsk) (Figure 5C–F) were also significantly upregulated. Although our scaffold‐mediated delivery approach provided localized miR delivery, these biomolecules were still taken up by cells in a non‐specific/non‐targeted manner. Hence, in attempt to understand the potential mechanisms associated with Axon miR treatment, we focused on the 62 significantly regulated genes in Axon miRs + Mp‐treated rats versus Neg miR + Mp‐treated rats (Figure S13, Supporting Information). Since the rat SCI complete transection injury may also result in other non‐neural cells infiltrating into the site of injury and bulk RNA‐sequencing does not provide cell‐specific information, we referred to the Brain RNA‐seq dataset^[^ ^41^ ^]^ and shortlisted genes (FPKM > 10) that have been found to be abundant in neurons and glial cells within the CNS. Correspondingly, Axon miRs treatment appear to 1) stabilize the ECM and prevent matrix degradation (increased Hapln1 (Hyaluronan And Proteoglycan Link Protein 1) expression;^[^ ^42^ ^]^ and decreased CCL7 expression); 2) reduce proinflammatory responses (increased Fkbp5 expression, which suppresses the immune reaction;^[^ ^43^ ^]^ and decreased Ptx3,^[^ ^44^ ^]^ CD74,^[^ ^45^ ^]^ CCL7^[^ ^46^ ^]^ and CXCL1^[^ ^47^, ^48^ ^]^ expressions, which are pro‐inflammatory related genes); 3) affect JUN family protein interactions by decreasing JUN family involvement (increased JDP2 expression, which represses transactivation mediated by Jun family of proteins;^[^ ^49^ ^]^ and decreased FOS and FOSB expressions);^[^ ^50^, ^51^ ^]^ and 4) promote OPC and OL proliferation and survival response by increasing IGFBP3 expression.^[^ ^52^ ^]^

While these observations appear promising and in agreement with the outcomes of our other experiments, the bulk RNA‐seq analyses did not reveal significant knockdown of the downstream targets of the Axon miRs. One possible reason may be that the effects of miRs were masked by glial cells, which exist in much larger quantities versus neurons. The spinal cord possesses about 1.5–1.7 billion cells, of which ≈13.4% are neurons, whereas the remaining 12.2% and 74.8% are endothelial and glial cells respectively. Hence, the glia: neuron ratio is around 5.6–7.1.^[^ ^53^ ^]^ Given this possible masking of results, analyses at the single cell level, such as single cell transcriptomics using single cell RNA‐sequencing, can reveal cell heterogeneity and should be employed in future studies to further elucidate the effects of Axon miRs in SCI treatment.

Altogether, the novel findings of this work lie not in the use of methylprednisolone, but in its effects when administered along with nerve regeneration‐enhancing miRs. These novel findings include 1) administration of both Axon miRs and methylprednisolone reduced cyst formation, decreased inflammatory response, and increased ECM‐related expression. 2) Significant improvements in the rate and extent of sensory function recovery, as well as, the extent of motor function recovery were observed when Axon miRs were further increased.

The fiber‐hydrogel scaffold includes 1) Polycaprolactone (PCL) electrospun fibers; 2) Collagen matrix; 3) Growth factors; 4) Heparin; 5) miRs with cationic transfection reagent TKO; 6) Methylprednisolone. The solid structures that made up the fiber‐hydrogel scaffold are PCL electrospun fibers and collagen matrix, both of which are Food and Drug Administration (FDA) approved biodegradable materials. PCL has been extensively investigated as injectable and implantable biomaterials for controlled release drug delivery systems.^[^ ^54^, ^55^, ^56^ ^]^ Type 1 collagen is the most abundant constituent in the extra cellular matrix of living tissue^[^ ^57^ ^]^ and has been frequently used in creating 2D and 3D environment for cell proliferation, migration, as well as nerve tissue growth.^[^ ^58^, ^59^, ^60^, ^61^ ^]^ For heparin and methylprednisolone, both are FDA approved drugs which have been used in clinical settings. Additionally, FDA has approved two recombinant human growth factors for clinic usage in 1991 and 1997, namely, recombinant human‐GM‐CSF (rh‐GM‐CSF)^[^ ^62^, ^63^ ^]^ and recombinant human‐PDGF (rh‐PDGF‐BB).^[^ ^64^, ^65^, ^66^ ^]^ With these two growth factors approved, we believe the approval of GDNF for use clinically will not present a major hurdle. Finally, the recent Covid‐19 vaccine based on messenger RNA (mRNA) technology suggests that gene therapy, which includes miRs, may, in future, be increasingly accepted. Therefore, we believe that our approach holds great potential in clinical translationability.

## 4 Conclusion

In this study, we applied a 3D fiber‐hydrogel scaffold to deliver Axon miRs to the transected rat spinal cord non‐virally. Within the dosages used, Axon miRs significantly enhanced mature axon regeneration. Additionally, in the presence of methylprednisolone, the effects of Axon miRs in enhancing mature axon regeneration remained unaffected. More importantly, Axon miRs in the presence of methylprednisolone reduced cyst formation and provided a trend of improved functional recovery. Further analyses by bulk RNA‐sequencing suggest that treatment with Axon miRs in the presence of methylprednisolone decreased inflammatory response and increased ECM deposition. By doubling the amount of Axon miRs, we saw significant improvements in the rate and extent of sensory function recovery, as well as, the extent of motor function recovery without alteration in axon regeneration. Besides that, the myelination index of boosted Axon miRs‐treated rats (i.e., 10 µg) was also significantly enhanced, especially outside the scaffolds. Altogether, SCI treatment using scaffold‐mediated delivery of Axon miRs, in the presence of methylprednisolone, is a promising therapeutic approach.

## 5 Experimental Section

### Materials

PCL (molecular weight 45 and 80 kDa), 2,2,2‐trifluoroethanol (TFE, ≥99.0%), Dimethyl formamide (DMF, ≥99.0%), poly‐d‐Lysine (PDL) (P0899), heparin sodium, and DNAse were purchased from Sigma‐Aldrich. Alexa‐Fluor 488 goat anti‐mouse, Alexa‐Fluor 555 goat anti‐Mouse, Alexa‐Fluor 488 goat anti‐rabbit, Alexa‐Fluor 555 goat anti‐rabbit, Alexa‐Fluor 555 goat anti‐chicken, scrambled negative miR (Neg miR), miR‐132‐3p (PM10166), miR‐222‐3p (PM11376), and miR‐431‐5p (PM10091), DAPI (4′,6‐diamidino‐2‐phenylindole), paraformaldehyde (PFA, 7 230 681), 10x phosphate buffered saline (PBS; pH7.4), goat serum, neurobasal medium, bovine serum albumin (BSA, A1000801), Fluoromount‐G (00‐4958‐02), and Quant‐iT RiboGreen RNA reagent kit (Invitrogen) were obtained from Life Technologies. TransIT‐TKO Transfection Reagent was purchased from MirusBio. Mouse anti‐*β*III Tubulin (Tuj‐1) (801 202) and chicken anti‐NF200 (822 601) were purchased from Biolegend. Rat anti‐MBP antibody (aa82‐87) was purchased from Bio‐Rad. Rabbit anti‐GFAP (Z0334) was obtained from DAKO. Rabbit anti‐5HT was obtained from cell signaling technology. Rabbit anti‐Iba1 (019‐19741) was procured from Fujifilm Wako Chemicals USA Corporation. Mouse anti‐Calcitonin gene‐related peptide (CGRP, sc‐57053) was purchased from Axil Scientific. Mouse anti‐iNOS (610 328) was bought from BD Biosciences. Rabbit anti‐synaptophysin (Syn, ab32127) was purchased from Abcam. Rat‐tail collagen type I was ordered from Corning. GDNF was procured from PeproTech. GDNF ELISA kit was purchased from Singlab Technologies. RNA extraction kit was obtained from Qiagen. Methylprednisolone was purchased from Allpets Asia Pte Ltd.

### Fiber‐Hydrogel Scaffold Fabrication and Characterization—Fiber‐Hydrogel Scaffold Fabrication

Fiber‐hydrogel scaffolds were fabricated following our established protocols.^[^ ^16^, ^67^ ^]^ Briefly, PCL was dissolved overnight in TFE at 14% w/w before use to ensure homogeneity. A two‐pole airgap electrospinning technique was adopted to fabricate aligned PCL fibers. Correspondingly, the electrospinning solution was loaded into a 3 mL syringe that was subsequently capped with a 21‐gauge needle. This needle tip was then charged at positive 8 kV and the two‐pole airgap collector was charged at negative 4 kV. The electrospinning solution was extruded at a flow rate of 2.5 mL h^−1^ using a syringe pump and the electrospun fibers were deposited between the two stationary poles spaced 5 cm apart. A complete fiber stack was obtained after 4 min of continuous spinning. These fiber stacks (i.e., 11 layers) were then sterilized for 30 min using UV irradiation before subsequent steps. Alternate fiber layers were pre‐wetted with 70% ethanol (5 min x 2), then washed with distilled water (5 min x 3) before coating with PDL (100 µg mL^−1^, 1 mL per fiber layer) overnight at 37 °C. Following PDL coating, the fiber stacks were lyophilized, piled up in alternate fashion with the uncoated fiber layers, and rolled into a bundle (11 fiber stacks per bundle). Subsequently, the fiber bundle was positioned centrally within a sterilized cylindrical mold (10 mm in length and 3.5 mm in inner diameter) prior to the addition of collagen.

Rat‐tail type 1 collagen was used to form the hydrogel matrix surrounding the fiber bundle according to the manufacturer's protocol. Briefly, 10x PBS, 1N NaOH, deionized (DI) water and collagen were added into a sterile 600 µL microtube in the listed order and gently mixed to obtain a 300 µL collagen solution (volume used to completely fill the 10 mm cylindrical mold) with a final concentration of 3.5 mg mL^−1^. Additionally, GDNF was added to promote axonal ingrowth into the scaffold. Fresh GDNF powder was reconstituted in 0.1% BSA and 400 µg µL^−1^ heparin at 1:1 v/v ratio to obtain a stock concentration of 2 µg µL^−1^. Following that, 12.5 µL of the GDNF stock solution (25 µg GDNF) was used to substitute 12.5 µL of DI water in the 300 µL collagen solution. Heparin was used with GDNF because it functions to activate growth factors.^[^ ^68^, ^69^, ^70^ ^]^ Specifically, heparin and heparan sulfate proteoglycans bind many soluble growth factors, which enhances the formation of high‐affinity complexes between the growth factors and their receptors.^[^ ^71^ ^]^ Besides that, heparin could also prevent the proteolysis of growth factors,^[^ ^72^, ^73^, ^74^ ^]^ thus serving as a storage depot which increases tissue growth factor levels and activities. For low dose miR loaded scaffolds, an additional 25 µg of miRs consisting of miR‐132, miR‐222, and miR‐431 (Axon miRs) at a 1:1:1 v/v ratio (the three miRs were utilized in equal concentration) were complexed with TKO (miR: TKO 1:1 v/v ratio) and used for replacing an equivalent volume of DI water in the collagen mixture. A total of 5 µg of GDNF and 5 µg of miRs were used per animal. On the other hand, 50 µg of Axon miRs were complexed with TKO and loaded in the collagen mixture in high dose miR‐loaded scaffolds with the same amount of GDNF. A total of 5 µg of GDNF and 10 µg of miRs were used per animal. The miR loaded collagen mixture was then dispensed into the mold surrounding the electrospun fibers and allowed to solidify into a gel at room temperature for 30 min. Following that, the scaffold was transferred to a minus 20 °C freezer for 4 h before being lyophilized overnight. The scaffold fabrication process is illustrated in Figure 1A. Scaffolds were cut into 2 mm in length under sterile conditions before implantation.

### Scaffold Morphology and Fiber Diameter Assessment

The morphology of the scaffold was evaluated by scanning electron microscopy (SEM) (JEOL, JSM‐6390LA, Japan) under an accelerating voltage of 10 kV after sputter‐coating with platinum for 100 s at 10 mA. The average fiber diameter was quantified by measuring 100 fibers from high magnification images (2500x) using Image J software (NIH, USA).

### Drugs Release Kinetics

To obtain drug loading efficiencies and release profiles, each fiber‐hydrogel scaffold (*n* = 3) containing Neg miR complexes and GDNF was completely submerged into 0.5 mL of 1x PBS and incubated at 37 °C. At each time point, 0.5 mL of supernatant was collected and an equal volume of fresh PBS was added. The amount of GDNF released over time was detected using a GDNF ELISA kit. For the quantification of miRs, 4 µL of heparin (100 mg mL^−1^) was added to 100 µL of each tube of supernatant and thoroughly mixed for 15 min to decomplex Neg miR from TKO. The concentration of miRs present in the supernatant was then quantified using Quant‐iT RiboGreen assay following the manufacturer's protocol. At the end of the release kinetics study, all scaffolds were further digested with 300 µL of collagenase type 1 (100 U mL^−1^) for 1 h at 37 °C to extract all remaining GDNF and miRs, which were subsequently quantified using the methods described above. The loading efficiency of miRs and GDNF were computed using the equation stated below:

(1) $$\begin{array}{ccc} & & \mathrm{Loading}\;\mathrm{efficiency}\left(\%\right)\\ & & \;=\;\frac{\mathrm{Total}\;\mathrm{mass}\;\mathrm{of}\;\mathrm{drugs}\;\mathrm{released}\left(\mathrm{ng}\right)+\mathrm{Total}\;\mathrm{mass}\;\mathrm{of}\;\mathrm{drugs}\;\mathrm{extracted}\left(\mathrm{ng}\right)}{\mathrm{Total}\;\mathrm{theoretical}\;\mathrm{mass}\;\mathrm{of}\;\mathrm{drugs}\;\mathrm{loaded}\left(\mathrm{ng}\right)}\\ & & \;\times \;100\% \end{array}$$

The cumulative release profiles were plotted as a percentage of the experimental mass of GDNF and miRs that were loaded into the scaffolds.

### Fabrication and Characterization of Methylprednisolone‐Laden Electrospun Nanofiber Mats

Methylprednisolone (50 mg, maximum solubility) was dissolved in a TFE/DMF mixed solution (9:1 v/v, 500 µL) containing a PLA‐Pluronic copolymer (i.e., PLA50‐P127, Mw 100 kDa, named as 50P100, 20 wt%). 50P100 was synthesized following the published protocol.^[^ ^75^ ^]^ The obtained solution was loaded into a syringe and dispensed at a fixed rate (1.0 mL h^−1^) by a syringe pump. Voltages of +8 kV and −4 kV were then applied to the blunt needle tip and the collector, respectively. A 4 cm × 4 cm aluminum foil was used for the collection of methylprednisolone‐laden electrospun nanofiber mats, and the distance between spinneret and collector was set as 20 cm. The obtained electrospun nanofiber mats were then sterilized by UV irradiation for 30 min. Thereafter, the surface morphology of the mats was observed under a SEM (JEOL, JSM‐6390LA) at a 10 kV accelerating voltage. To measure the release profile of methylprednisolone, the obtained electrospun nanofiber mats (2 mm × 10 mm) were immersed in PBS (1 mL) and incubated at 37 °C. At each time point, 1 mL of PBS was collected from each sample and replaced with an equal volume of fresh PBS. The supernatant was then used to measure the amount of released methylprednisolone by UV–vis (Shimadzu UV‐2450). PLA is more hydrophilic and degrades much faster than PCL,^[^ ^76^ ^]^ therefore it facilitated the release of methylprednisolone from the mat. The morphology of the electrospun mats, release profile based on the absolute amount as well as the percentage cumulative release based on experimental loading are shown in Figure S2, Supporting Information.

To derive the dosage of methylprednisolone for localized delivery, an approximation was conducted. Specifically, rats have around 64 mL of blood per kg of bodyweight,^[^ ^77^ ^]^ according to the guideline for rodent survival blood collection. The SD rats utilized in our experiments weigh around 250 g, which corresponds to around 16 mL of blood per rat. For 30 mg kg^−1^ systemic delivery of methylprednisolone, there is ≈469 µg methylprednisolone present in 1 cm^3^ of blood (469 µg cm^−3^ of blood). For spinal cord transection injury, the volume of blood pooling within the space is ≈1 cm^3^ as well. Therefore, the amount of methylprednisolone to be encapsulated within each implanted mat was determined to be 500 µg. This value is exactly 1/15 of the amount of methylprednisolone administered intravenously in our rats (7.5 mg/250g rat). This ratio is also in good agreement with a previous work which delivered methylprednisolone locally (1/20) using PLGA nanoparticles for treating spinal cord injured rats.^[^ ^78^ ^]^ Importantly, the amount of methylprednisolone released from the mat within 24 h was quantified to be around 144.1 µg mg^−1^ (Figure S2, Supporting Information). The average mass of each implanted mat was 3.25 mg and hence the amount of methylprednisolone released from the mat is 468 µg, which is very similar to the 469 µg approximated. Therefore, the low and high dose Axon miR‐treated animals are comparable even though the dosing regimen of methylprednisolone is different.

### Spinal Cord Transection and Scaffold Implantation

The animal care and experimental procedures were carried out in accordance with the Institutional Animal Care and Use Committee guidelines of Nanyang Technological University (IACUC, NTU, Protocol number A0309). Animals were housed under temperature‐controlled conditions, with a normal 12/12 h light/dark cycle with ad libitum access to water and food. Female Sprague‐Dawley rats (8–9 weeks, 200–250 g) were obtained from In Vivos Pte Ltd (Singapore). The rats were anesthetized via intraperitoneal injection of ketamine and xylazine (0.2 mL per 100 g of weight). The surgical field was shaved, cleaned with 70% ethanol and treated with betadine. Thereafter, the skin was incised above the thoracic level, and the muscles were moved apart to expose the vertebra at level T8–T11. A dorsal laminectomy was performed on T9–T10. Following that, the dura was opened and 2 mm of the spinal cord was removed using fine microscissors. A 2 mm long fiber‐hydrogel scaffold was then implanted snugly between the rostral and caudal stump of the transected spinal cord. Thereafter, the dura was sutured and a 3 mm by 3 mm PCL film with thickness of around 50 µm was placed above the spinal cord to cover the injury area. Following that, the muscles were sutured and the skin was closed with wound clips. Animals were randomly divided into eight groups as presented in Table S1a–d, Supporting Information. In the low dose Axon miR treated rats (i.e., 5 µg of Axon miRs) and samples used for RNA sequencing, a high dose of methylprednisolone (30 mg kg^−1^) was administrated via the jugular vein^[^ ^17^, ^79^, ^80^ ^]^ at 5 min, 2 h and 24 h post SCI. However, in the high dose Axon miR treated rats (i.e., 10 µg of Axon miRs), the delivery approach of methylprednisolone was changed to localized delivery via electrospun nanofiber mats due to the high mortality rate (i.e., ≈28%) that was induced by jugular vein injections in the low dose Axon miR treated rats. It has been revealed that localized delivery of therapeutics to the injured spinal cord could minimize the side effects associated with high‐dose systemic delivery while maximizing the therapeutic effects.^[^ ^78^, ^81^ ^]^ As such, instead of covering the site of injury with a plain PCL film, the wound site was covered with a 4 mm by 4 mm methylprednisolone‐loaded electrospun nanofiber mat (i.e., 545.4 µg) for the high dose Axon miR treated rats.

### RNA Sequencing of T9‐T11 Spinal Cord Segment

One week after surgery, rats with different treatments (Table S1a, Supporting Information, sham with methylprednisolone administered (Sham, 3 rats), injured rats without scaffold implantation but with methylprednisolone administered (SCI, 3 rats), injured rats treated with Neg miR incorporate scaffolds and methylprednisolone administration (Neg miR + Mp, 3 rats) and injured rats treated with Axon miRs incorporated scaffolds and methylprednisolone administration (Axon miRs + Mp, 3 rats) were sacrificed and their entire T9–T11 spinal cord segment, including the scaffold, were promptly harvested under a dissection microscope. RNA was then extracted with the RNeasy Mini kit (Qiagen), as per manufacturer's recommendations, and assessed for RNA integrity with the Agilent RNA 6000 Pico Kit. Extracted RNA from one SCI‐treatment rat and one Neg miR + Mp condition rat had low RNA integrity (RIN < 7.0) and were omitted from further analysis. 1 µg of total RNA from each rat was used for mRNA‐seq library preparation with the Illumina Truseq Stranded mRNA library prep kit. Prepared libraries were multiplexed and 151 bp paired end sequenced on one lane of the Illumina HiSeq4000 platform. All sequenced reads were aligned to the *Rattus norvegicus* genome (Rnor 6.0/rn6) with STAR version 2.5.2a^[^ ^82^ ^]^ and transcripts were assembled with RSEM version 1.3.0.^[^ ^83^ ^]^ Cuffnorm version 2.2.1^[^ ^84^, ^85^ ^]^ was used to obtain quartile normalized FPKM expression matrix of all genes across all samples (Sham, SCI, Neg miR + Mp, and Axon miRs + Mp).

### Hierarchical Sample Clustering

The normalized gene expression matrix was utilized to construct a hierarchical clustering dendrogram using R package cluster version 2.0.7.1 (https://cran.r‐project.org/web/packages/cluster/index.html) and dendextend version 1.10.0 (https://cran.r‐project.org/web/packages/dendextend/index.html).

### Differential Gene Expression Analysis

Cuffdiff2 version 2.2.1^[^ ^84^, ^85^ ^]^ was used to identify differentially expressed genes (DEGs) between samples. Significant DEGs have false discovery rate (*q*) ≤0.05 and FPKM ≥ 1 in at least one sample group in the comparison. The Kyoto Encyclopedia of Genes and Genomes database (KOBAS 3.0) was used to identify enriched pathways in DEGs relatively to the whole genome background using default settings.^[^ ^86^ ^]^ The rich factor of pathway enrichment was calculated using the number of significantly regulated genes in a specific pathway divided by the number of all background genes in that pathway.

### Correlation Analysis

Correlation analysis of Sham, SCI, Neg miR + Mp, and Axon miRs + Mp transcriptome profiles was conducted by calculating Pearson correlation coefficient of each sample pair with the cor R function. The obtained correlation matrix was then visualized in a correlogram with the corrplot R package (version 0.84).

### Basso, Beattie, and Bresnahan Scoring

The hindlimb recovery of animals was evaluated through a weekly open field test using the Basso, Beattie, and Bresnahan scoring system.^[^ ^87^ ^]^ Briefly, animals were placed within a circular enclosure (diameter of 1.5 m) and allowed to roam around freely. Each rat was filmed for a duration of 3 min and their scores were assessed by two independent observers blinded to group identity. Specifically, to avoid the risk of scoring spastic responses or spontaneous twitches, a response was only scored when it occurred three times during the 3 min observation period.^[^ ^36^ ^]^

### Von Frey Hair Test

The rats’ hind paw withdrawal threshold was determined using the Von Frey hair test^[^ ^88^ ^]^ and expressed in grams. Briefly, monofilaments, which required increasing amounts of buckling force ranging from 0.23 to 59 g, were applied perpendicularly to the plantar surface of the hind paw until a response was observed. A response is considered positive if the animal exhibits any nocifensive behaviors, including brisk paw withdrawal, licking, or shaking of the paw, either during application of the stimulus or immediately after the filament was removed.^[^ ^88^ ^]^ Three measurements were obtained from each animal and the interval between each test was not less than 1 min. If two positive responses were obtained within the three measurements, a thinner filament will be applied. Otherwise, a thicker filament would be applied. If the animal responded well to the thicker filament, the previous thinner filament would be applied again. This process was repeated until two positive results for a particular filament was obtained twice. The buckling force of that filament was then assigned as the paw withdrawal threshold for the corresponding animal.

### Biotinylated Dextran Amine Retrograde Tracing

Following the weekly behavior tests until week 12, animals in each treatment group were randomly separated into two batches. In one batch of animals, BDA was injected at 5 mm rostral to the injured area while in the other batch, BDA was injected at 5 mm caudal to the injured region. Briefly, animals were anesthetized and placed under the surgical microscope. The spine and dura were then excised and 1 µL of BDA (BDA 3000 MW, 10% in PBS, pH 7.4, Thermo Fisher) was injected at a depth of 2 mm on both sides of the spinal cord laterally. Animals were kept for another week before being sacrificed.

### Immunohistochemical Analysis

At the predetermined time points, animals were perfused with 0.9% saline followed by 4% PFA. After perfusion, spinal cords containing the implanted scaffold were retrieved and post fixed with 4% PFA for another 24 h before transferring into 15% sucrose for 24 h. Thereafter, samples were transferred to 30% sucrose and stored at 4 °C until sectioning. Spinal cord samples were sectioned longitudinally at 20 µm thickness using a cryostat (Leica CM1950) and directly mounted onto glass slides. Immunofluorescent staining was performed to evaluate the effects of the treatment on various cell types of interest. Briefly, the frozen sections were permeabilized with 0.3% TritonX‐100 for 15 min before being incubated in blocking buffer (10% goat serum) for at least 1 h. Primary antibodies were then added and incubated at 4 °C overnight. The following primary antibodies were used: mouse anti‐Tuj1 (1:1000 dilution), chicken anti‐NF200 (1:1000 dilution), rabbit anti‐GFAP (1:1000 dilution), mouse anti‐CGRP (1:500 dilution) and rabbit anti‐5‐HT (1:1000 dilution), rat anti‐MBP (1:200 dilution), rabbit anti‐synaptophysin (Syn, 1:200 dilution), rabbit anti‐Iba1 (1:500 dilution), and mouse anti‐iNOS (1:100 dilution). Samples were subsequently washed three times with PBS and incubated with the following secondary antibodies for 2 h at room temperature: Alexa Fluor 555‐conjugated goat anti‐chicken (1:500 dilution), Alexa Fluor 488‐conjugated goat anti‐rat (1:500), Alexa Fluor 555‐conjugated goat anti‐mouse (1:500), and Alexa Fluor 488‐conjugated goat anti‐rabbit (1:500 dilution). To visualize BDA, avidin‐488 (1:500 dilution) was used to stain the samples for 2 h at room temperature. Nuclear staining was performed by incubating the sections with DAPI (1:1000 dilution) at room temperature for 10 min after the secondary antibodies. Thereafter, all sample slides were coverslip mounted using Fluoromount‐G.

For axonal ingrowth quantification (NF200 and Tuj1 positive signals), stitched images of the injury site were taken under 10x magnification using a fluorescence microscope (Leica DMi8). The percentage of neurofilament or tubulin staining that was identified in the scaffold was quantified (three spinal cord sections per animal) using ImageJ software (NIH, USA) based on the following equations:

(2) $$\begin{array}{ccc} & & \mathrm{Area}\;\mathrm{percent}\;\mathrm{of}\;\mathrm{NF}200\;\mathrm{in}\;\mathrm{scaffold}\left(\%\right)\\ & & \;=\;\frac{\mathrm{Area}\;\mathrm{occupied}\;\mathrm{by}\;\mathrm{NF}200^{+}\;\mathrm{pixels}\left(\mu m^{2}\right)}{\mathrm{Area}\;\mathrm{of}\;\mathrm{scaffold}\left(\mu m^{2}\right)}\times 100\% \end{array}$$

(3) $$\begin{array}{ccc} & & \mathrm{Area}\;\mathrm{percent}\;\mathrm{of}\;\mathrm{Tuj}1\;\mathrm{in}\;\mathrm{scaffold}\left(\%\right)\\ & & \;=\;\frac{\mathrm{Area}\;\mathrm{occupied}\;\mathrm{by}\;\mathrm{Tuj}1^{+}\mathrm{pixels}\left(\mu m^{2}\right)}{\mathrm{Area}\;\mathrm{of}\;\mathrm{scaffold}\left(\mu m^{2}\right)}\times 100\% \end{array}$$

The quantification method for BDA, 5‐HT, and CGRP staining was exactly as described for both NF200 and Tuj1 staining. The only difference was that the images for quantifying BDA were taken at 20x magnification using a fluorescence microscope (Leica DMi8) while the images for quantifying 5‐HT and CGRP were taken at 20x magnification under a confocal microscope (Zeiss LSM 800). Accordingly, the percentage of BDA^+^ signals within the scaffold was quantified. Besides that, the percentage of 5‐HT^+^ and CGRP^+^ signals that was identified in rostral region, caudal region, and within the scaffold were quantified respectively using ImageJ software (NIH, USA) based on the following equations. At each region, three ROIs were imaged per section and three spinal cord sections were included per animal.

(4) $$\begin{array}{ccc} \mathrm{Area}\;\mathrm{percent}\;\mathrm{of}\;X\;\mathrm{in}\;\mathrm{scaffold}\left(\%\right) & = & \frac{\mathrm{Area}\;\mathrm{occupied}\;\mathrm{by}\;X^{+}\;\mathrm{pixels}\;\left(\mu m^{2}\right)}{\mathrm{Area}\;\mathrm{of}\;\mathrm{scaffold}\;\left(\mu m^{2}\right)}\\ & & \times \;100\% \end{array}$$

X Represents Biotinylated Dextran Amine, 5‐HT, and CGRP.

For glial scar measurement (3 spinal cord sections per animal), the percent area of GFAP^+^ signal within 500 µm from the interface of the injury site was quantified as previously reported.^[^ ^67^ ^]^ The GFAP^+^ signals were correlated to pixel intensity of the fluorescent images. The images were converted to 8‐bit and thresholded to segregate the GFAP^+^ signals from the background autofluorescence. All images were taken under the same microscopy settings and quantifications were conducted using ImageJ software (NIH, USA).^[^ ^16^, ^67^ ^]^

To quantify myelination index in the implant interface and within the scaffold, 40x magnified z‐stacks images obtained from Zeiss LSM800 confocal microscopy along with a colocalization software from ImageJ (NIH, USA) were used.^[^ ^16^, ^67^ ^]^ A total of three spinal cord sections were quantified per animal. At each region (i.e., rostral region, inside scaffold and caudal region), three ROIs were imaged per section for quantification. The myelination index is defined by the following equation:

(5) $$\begin{array}{ccc} & & \mathrm{Myelination}\;\mathrm{index}\left(\%\right)\\ & & \;=\frac{\mathrm{Number}\;\mathrm{of}\;\mathrm{overlapping}\;\mathrm{NF}200^{+}\;\mathrm{and}\;\mathrm{MB}P^{+}\;\mathrm{pixels}}{\mathrm{Number}\;\mathrm{of}\;\mathrm{NF}200^{+}\;\mathrm{pixels}}\;\times 100\% \end{array}$$

To quantify synaptic index within the scaffold, 20x magnified images obtained from Zeiss LSM800 confocal microscopy along with a colocalization software from ImageJ (NIH, USA) were used. A total of three spinal cord sections (3 ROIs were imaged per section) were used for quantification per animal. The synaptic index is defined by the following equation:

(6) $$\begin{array}{ccc} \mathrm{Synaptic}\;\mathrm{index}\left(\%\right) & = & \frac{\mathrm{Number}\;\mathrm{of}\;\mathrm{overlapping}\;\mathrm{Tuj}1^{+}\;\mathrm{and}\;\mathrm{Sy}n^{+}\;\mathrm{pixels}}{\mathrm{Number}\;\mathrm{of}\;\mathrm{Tuj}1^{+}\;\mathrm{pixels}}\\ & & \times \;100\% \end{array}$$

The abundance of microglial present in the implant interface and within the scaffold was quantified using images (40x magnification) obtained from a confocal microscope (Zeiss LSM 800). A total of three spinal cord sections (3 ROIs were imaged per section) were quantified per animal. The percentage of microglial and those expressing iNOS is defined by the following equations:

(7) $$\mathrm{Percent}\;\mathrm{of}\;\mathrm{Ib}a^{+}\mathrm{cells}\left(\%\right)=\frac{\mathrm{Number}\;\mathrm{of}\;\mathrm{DAP}I^{+}\mathrm{Ib}a^{+}\mathrm{cells}}{\mathrm{Total}\;\mathrm{number}\;\mathrm{of}\;\mathrm{DAP}I^{+}\mathrm{cells}}\;\times 100\%$$

(8) $$\begin{array}{ccc} \mathrm{Percent}\;\mathrm{of}\;\mathrm{Ib}a^{+}\mathrm{iNO}S^{+}\mathrm{cells}\left(\%\right) & = & \frac{\mathrm{Number}\;\mathrm{of}\;\mathrm{DAP}I^{+}\mathrm{Ib}a^{+}\mathrm{iNO}S^{+}\mathrm{cells}}{\mathrm{Total}\;\mathrm{number}\;\mathrm{of}\;\mathrm{DAP}I^{+}\mathrm{Ib}a^{+}\mathrm{cells}}\\ & & \times \;100\% \end{array}$$

The spinal cord sections used for quantifications were about 200 µm in distance from each other.

### Statistical Analysis

Outlier analysis was performed to exclude the outliers using GraphPad QuickCalcs software before any statistical analysis was done. Data were tested for normality and homogeneity of variances using the Shapiro‐Wilk test and Levene's test, respectively. For data which included comparison between two groups, student's *t*‐test was used. For data including comparison among three or more groups, one‐way ANOVA (parametric) followed by Bonferroni post‐hoc test was used. Otherwise, Kruskal–Wallis test (non‐parametric) followed by Mann–Whitney post‐hoc test was used. Statistical analysis was done using SPSS software. All values, unless mentioned otherwise, were represented as mean ± S.D.

## Conflict of Interest

The authors declare no conflict of interest.

## Supporting information

Supporting Information

## Data Availability

Research data are not shared.
