# Supplementary material for: A 3D Fiber‐Hydrogel Based Non‐Viral Gene Delivery Platform Reveals that microRNAs Promote Axon Regeneration and Enhance Functional Recovery Following Spinal Cord Injury
Source: Adv Sci (Weinh). 2021 May 29;8(15):2100805. doi: 10.1002/advs.202100805 (PMC8336488; doi:10.1002/advs.202100805)
Supplement: Supplementary file 1 — Supporting Information [file ADVS-8-2100805-s001.pdf]

© 2021 Wiley-VCH GmbH

ADVANCED SCIENCE
Open Access

Supporting Information

for *Adv. Sci.*, DOI: 10.1002/advs.202100805

A Three-dimensional Fiber-hydrogel based Non-viral Gene Delivery Platform Reveals that Micrornas Promote Axon Regeneration and Enhance Functional Recovery following Spinal Cord Injury

*Na Zhang#, Junquan Lin#, Vincent Po Hen Lin, Ulla Milbreta, Jiah Shin Chin, Elaine Guo Yan Chew, Michelle Mulan Lian, Jia Nee Foo, Kunyu Zhang, Wutian Wu and Sing Yian Chew**

Supplementary Tables:

Table. S1a. Description of *in vivo* treatment and sample size (RNA_seq)

Samples for RNA_seq	Description	Experimental duration	Sample size
Sham surgery + methylprednisolone	Spine and dura was opened but no injury was made. Methylprednisolone was administered via jugular vein.	1 week	3
Spinal cord transection + methylprednisolone	Spinal cord was transected but no scaffold was implanted. Methylprednisolone was administered via jugular vein.	1 week	2
Neg miR + methylprednisolone	Neg miR loaded scaffold was implanted into the transected spinal cord. Methylprednisolone was administered via jugular vein.	1 week	2
Axon miRs + methylprednisolone	Axon miRs loaded scaffold was implanted into the transected spinal cord. Methylprednisolone was administered via jugular vein.	1 week	3

Table. S1b. Description of *in vivo* treatment and sample size (Week 4)

Week 4	Description	Experimental duration	Sample size
Neg miR + methylprednisolone	Neg miR loaded scaffold was implanted into the transected spinal cord. Methylprednisolone was administered via jugular vein.	4 weeks	4
Axon miRs + methylprednisolone	Axon miRs loaded scaffold was implanted into the transected spinal cord. Methylprednisolone was administered via jugular vein.	4 weeks	4

Table. S1c. Description of *in vivo* treatment and sample sizes (Week 12)

Groups	Description	Experimental duration	Sample size
Neg miR + methylprednisolone	Neg miR loaded scaffold was implanted into the transected spinal cord. methylprednisolone was administered via jugular vein.	12 weeks	8
Axon miRs + methylprednisolone	Axon miRs loaded scaffold was implanted into the transected spinal cord. methylprednisolone was administered via jugular vein.	12 weeks	6
Boosted Axon miRs + methylprednisolone	Scaffold loaded with higher amounts of Axon miRs was implanted into the transected spinal cord. methylprednisolone was administered in a sustained fashion locally using an electrospun fibrous mat.	12 weeks	4
Neg miR	Neg miR loaded scaffold was implanted into the transected spinal cord.	12 weeks	10
Axon miRs	Axon miRs loaded scaffold was implanted into the transected spinal cord.	12 weeks	10

Table. S1d. Description of *in vivo* treatment and sample sizes (Week 2)

Week 2	Description	Experimental duration	Sample size
Untreated	Plain scaffold was implanted into the transected spinal cord.	2 weeks	4
GDNF	GDNF loaded scaffold was implanted into the transected spinal cord.	2 weeks	3

Supplementary Figures:

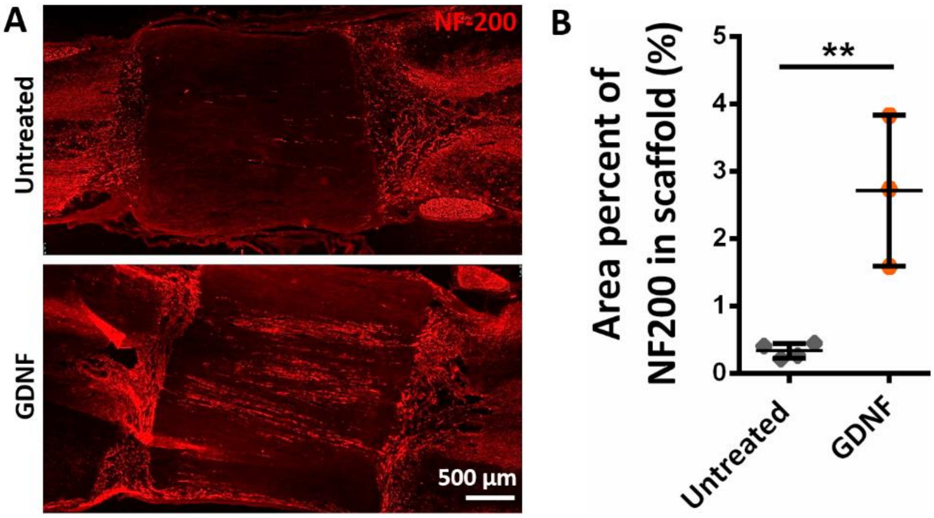

Figure S1. The administration of GDNF significantly promoted axon regeneration after SCI. (A) Representative fluorescent images of NF-200 expression inside the scaffold in Untreated and GDNF-treated samples at 2 weeks post SCI. (B) Quantification analysis of area percent occupied by $NF200^+$ signals within the scaffold region in Untreated and GDNF-treated rats. Results indicate that treatment with GDNF significantly enhanced nerve regeneration. **: $p < 0.01$, Student's t-test.

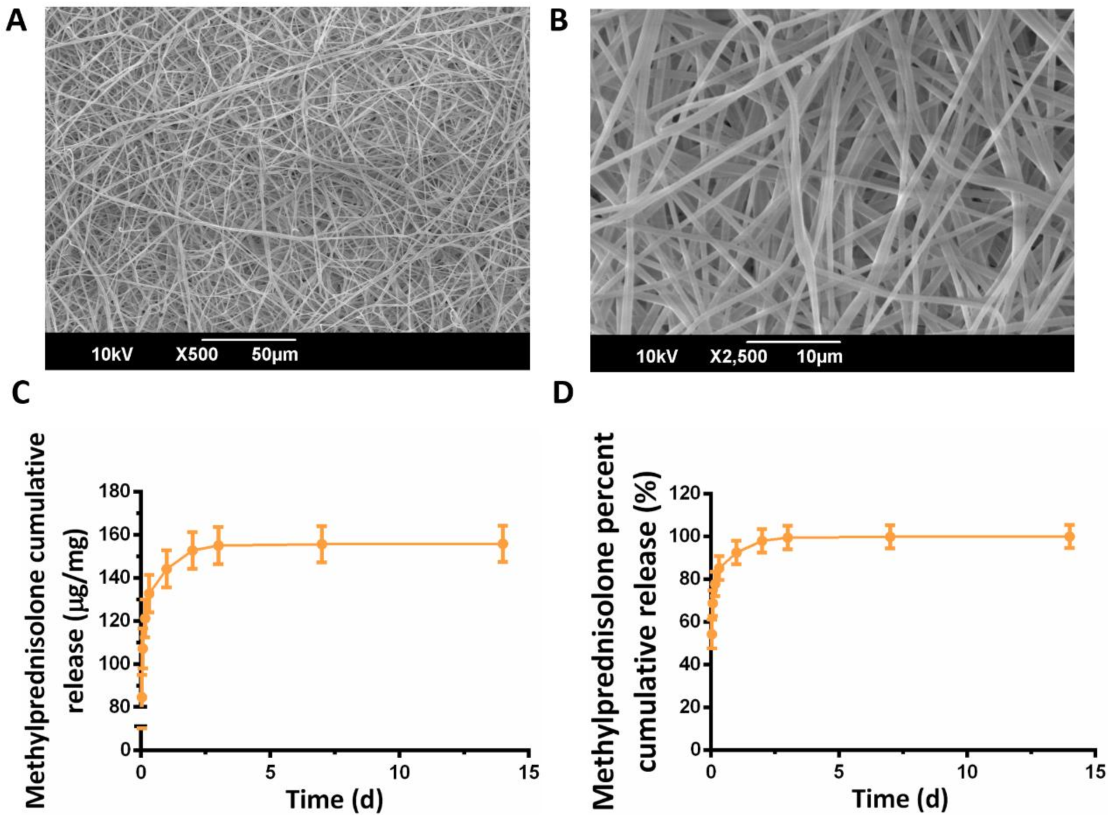

Figure S2. Characterizations of methylprednisolone-loaded 50P100 electrospun nanofiber mat. (A-B) SEM images of the methylprednisolone-loaded electrospun nanofiber mat. (C) Release profile of methylprednisolone from the electrospun nanofiber mat (expressed as mg/mg of scaffold). (D) Percent cumulative release based on experimental loading. All data are represented as mean ± SD.

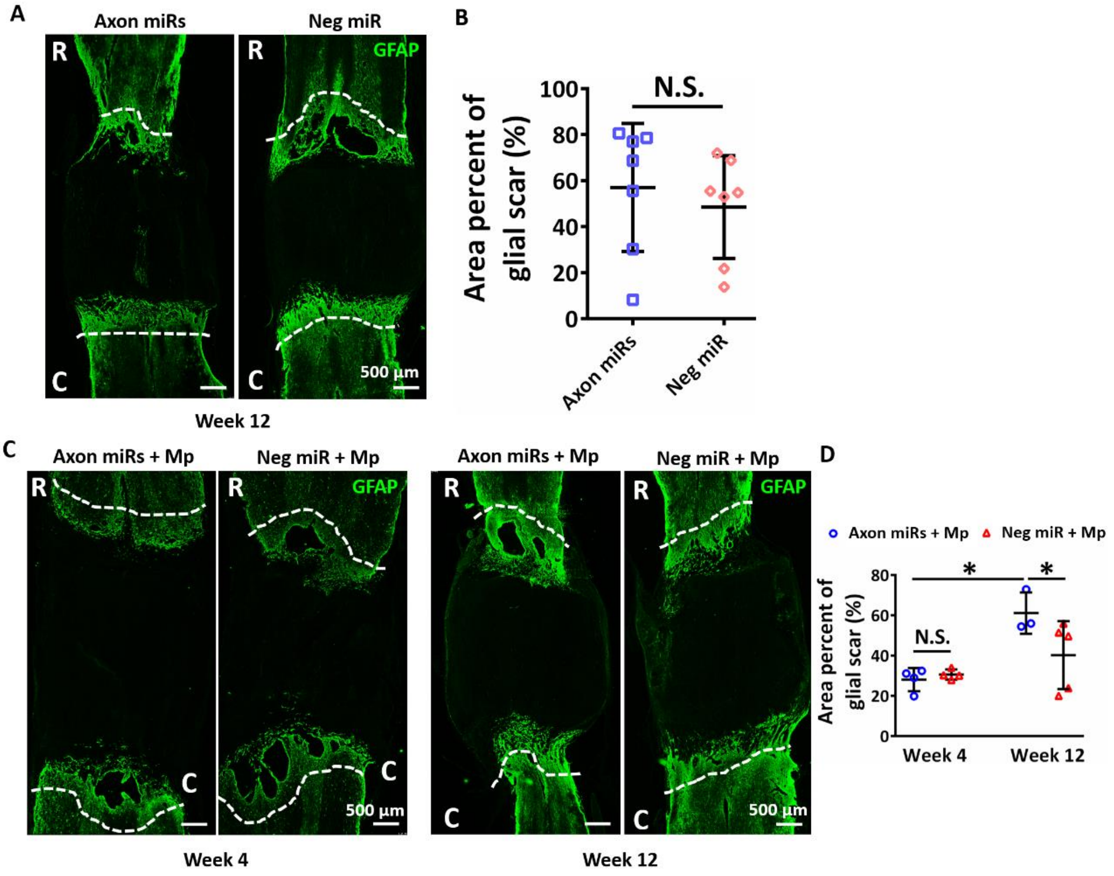

Figure S3. Administration of Axon miRs in the presence of methylprednisolone activated astrocytes at Week 12. (A) Representative fluorescent images of GFAP (green) expression at injury sites in Axon miRs- and Neg miR-treated rats at Week 12. (B) Quantification of area percent occupied by $GFAP^+$ signals from the tissue scaffold interface to 500 μm away from the interface (showed by the white dot line), suggesting that there is no significant difference in glial scar formation between Axon miRs- and Neg miR-treated rats at 12 weeks post SCI. Student's t-test. (C) Representative fluorescent images of GFAP (green) expression at injury sites in Axon miRs + Mp- and Neg miR + Mp-treated rats at both Week 4 and Week 12. (D) Quantification of area percent occupied by $GFAP^+$ signals. Result suggest that, in the presence of methylprednisolone, Axon miRs treatment increased the expression of GFAP significantly at Week 12 as compared to Neg miR control. *: $p < 0.05$, Shapiro-Wilk normality test followed by Kruskal-Wallis test and Mann-Whitney post hoc test. These results are not significantly different when compared with corresponding samples without methylprednisolone (Fig S2B and Fig S9). All data were represented as mean ± SD.

A

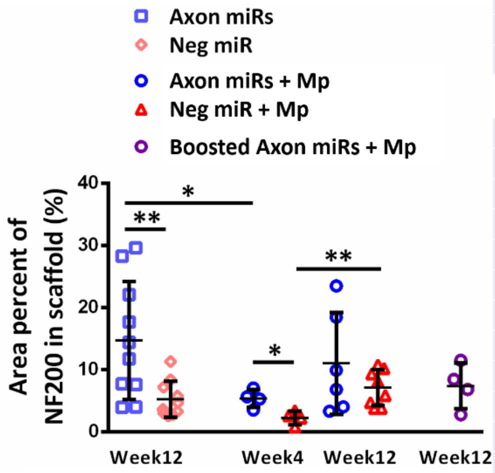

1	Week 12 Axon miRs
2	Week 12 Neg miR
3	Week 4 Axon miRs + Mp
4	Week 4 Neg miR + Mp
5	Week 12 Axon miRs + Mp
6	Week 12 Neg miR + Mp
7	Week 12 Boosted Axon miRs + Mp

	vs	p value			vs	p value	
1	2	0.007	**	4	5	0.019	*
	3	0.048	*		6	0.007	**
	4	0.005	**		7	0.043	*
	5	0.448	N.S				
	6	0.076			vs		
	7	0.157	N.S	5	6	0.606	N.S
					7	0.67	N.S
	vs						
2	3	0.671	N.S		vs		
	4	0.034	*	6	7	0.865	N.S
	5	0.104	N.S				
	6	0.11	N.S				
	7	0.258	N.S				
	vs						
3	4	0.021	*				
	5	0.394	N.S				
	6	0.308	N.S				
	7	0.386	N.S				

Figure S4. Combined analysis of $NF200^+$ axons in various groups at different time points. Data represented as mean ± SD., $*p < 0.05$; $**p < 0.01$, Shapiro-Wilk normality test followed by Kruskal-Wallis test and Mann-Whitney post hoc test.

A

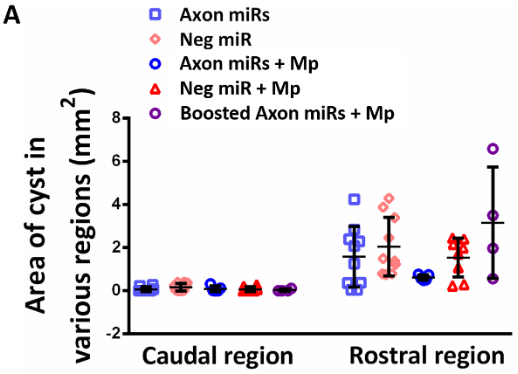

1	Week 12 Axon miRs Caudal
2	Week 12 Neg miR Caudal
3	Week 12 Axon miRs+ Mp Caudal
4	Week 12 Neg miR + Mp Caudal
5	Week 12 boosted Axon miRs + Mp Caudal
6	Week 12 Axon miRs Rostral
7	Week 12 Neg miR Rostral
8	Week 12 Axon miRs+ Mp Rostral
9	Week 12 Neg miR + Mp Rostral
10	Week 12 boosted Axon miRs + Mp Rostral

	vs	p value			vs	p value			vs	p value				p value	
1	2	0.15	N.S	2	3	0.383	N.S	3	4	0.524	N.S	4	5	0.613	N.S
	3	0.524	N.S		4	0.15	N.S		5	0.283	N.S		6	0.002	**
	4	1	N.S		5	0.122	N.S		6	0.01	**		7	0.0001	***
	5	0.613	N.S		6	0.008	**		7	0.002	**		8	0.003	**
	6	0.002	**		7	0.0001	***		8	0.009	**		9	0.001	***
	7	0.0001	***		8	0.002	**		9	0.008	**		10	0.005	**
	8	0.003	**		9	0.003	**		10	0.014	*				
	9	0.001	***		10	0.004	**								
	10	0.005	**												
	vs	p value			vs	p value			vs	p value			vs	p value	
5	6	0.011	*	6	7	0.364	N.S	7	8	0.003	**	8	9	0.143	N.S
	7	0.004	**		8	0.54	N.S		9	0.534	N.S		10	0.086	
	8	0.013	*		9	1	N.S		10	0.572	N.S				
	9	0.006	**		10	0.258	N.S								
	10	0.018	*												
	vs	p value													
9	10	0.308	N.S												

Figure S5. Combined analysis of cyst size in various groups at Week 12. Data represented as mean ± SD., *p < 0.05; **p < 0.01, Shapiro-Wilk normality test followed by Kruskal-Wallis test and Mann-Whitney post hoc test.

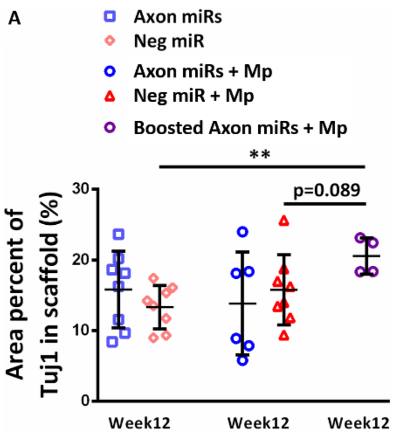

1	Week 12 Axon miRs
2	Week 12 Neg miR
3	Week 12 Axon miRs + Mp
4	Week 12 Neg miR + Mp
5	Week 12 Booste Axon miRs + Mp

	vs	p value			vs	p value			vs	p value	
1	2	0.248	N.S	2	3	1	N.S	3	4	0.519	N.S
	3	0.439	N.S		4	0.294	N.S		5	0.201	N.S
	4	1	N.S		5	0.007	**				
	5	0.174	N.S								
	vs	p value									
4	5	0.089									

Figure S6. Combined analysis of Tuj1^{+} axons in various groups at Week 12. Data represented as mean ± SD., *p < 0.05; **p < 0.01, Shapiro-Wilk normality test followed by Kruskal-Wallis test and Mann-Whitney post hoc test.

A Caudal injection

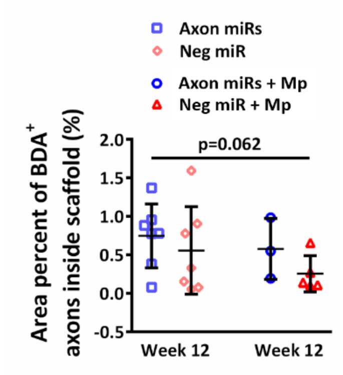

1	Week 12 Axon miRs
2	Week 12 Neg miR
3	Week 12 Axon miRs + Mp
4	Week 12 Neg miR + Mp

	vs	p value			vs	p value	
1	2	0.338	N.S	2	3	0.569	N.S
	3	0.732	N.S		4	0.465	N.S
	4	0.062					
	vs						
3	4	0.18	N.S				

B Rostral injection

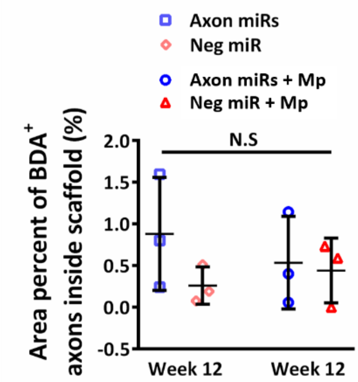

1	Week 12 Axon miRs
2	Week 12 Neg miR
3	Week 12 Axon miRs + Mp
4	Week 12 Neg miR + Mp

	vs	p value			vs	p value	
1	2	0.127	N.S	2	3	0.827	N.S
	3	0.513	N.S		4	0.513	N.S
	4	0.275	N.S				
	vs						
3	4	0.827	N.S				

Figure S7. Combined analysis of BDA$^+$ axons in various groups at Week 12. (A) Area percent of BDA+ axons when BDA was injected 5 mm below the injured region. (B) Area percent of BDA+ axons when BDA was injected 5 mm above the injured region. Data represented as mean ± SD., *p < 0.05; **p < 0.01, Shapiro-Wilk normality test followed by Kruskal-Wallis test and Mann-Whitney post hoc test.

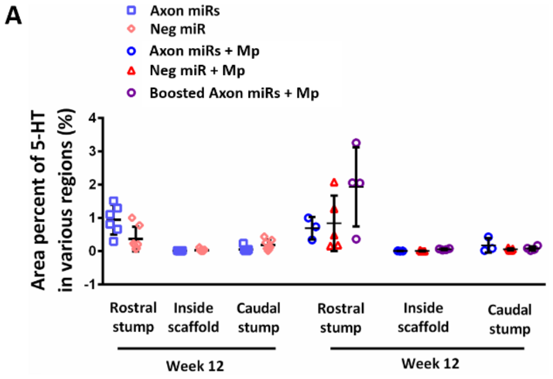

1	Week 12 Axon miRs Rostral
2	Week 12 Neg miR Rostral
3	Week 12 Axon miRs Inside
4	Week 12 Neg miR Inside
5	Week 12 Axon miRs Caudal
6	Week 12 Neg miR Caudal
7	Week 12 Axon miRs + Mp Rostral
8	Week 12 Neg miR + Mp Rostral
9	Week 12 Boosted Axon miRs + Mp Rostral
10	Week 12 Axon miRs + Mp Inside
11	Week 12 Neg miRs + Mp Inside
12	Week 12 Boosted Axon miRs + Mp Inside
13	Week 12 Axon miRs + Mp Caudal
14	Week 12 Neg miR + Mp Caudal
15	Week 12 Boosted Axon miRs + Mp Caudal

	vs	p value			vs	p value			vs	p value			vs	p value			vs	p value	
1	2	0.022	*	2	3	0.002	**	3	4	0.064		4	5	0.306	N.S	5	6	0.025	*
	3	0.003	**		4	0.004	**		5	0.018	*		6	0.013	*		7	0.017	*
	4	0.003	**		5	0.009	**		6	0.003	**		7	0.016	*		8	0.012	*
	5	0.003	**		6	0.406	N.S		7	0.017	*		8	0.004	**		9	0.008	**
	6	0.007	**		7	0.21	N.S		8	0.004	**		9	0.008	**		10	0.017	*
	7	0.439	N.S		8	0.291	N.S		9	0.008	**		10	0.084			11	0.042	*
	8	0.465	N.S		9	0.023	*		10	0.732	N.S		11	0.167	N.S		12	0.186	N.S
	9	0.136	N.S		10	0.017	*		11	0.685	N.S		12	0.088			13	0.17	N.S
	10	0.02	*		11	0.004	**		12	0.008	**		13	0.086			14	0.291	N.S
	11	0.006	**		12	0.023	*		13	0.017	*		14	0.122	N.S		15	0.155	N.S
	12	0.011	*		13	0.305	N.S		14	0.004	**		15	0.058					
	13	0.039	*		14	0.019	*		15	0.008	**								
	14	0.006	**		15	0.038	*												
	15	0.011	*																
	vs	p value			vs	p value			vs	p value			vs	p value			vs	p value	
6	7	0.053		7	8	0.881	N.S	8	9	0.221	N.S	9	10	0.034	*	10	11	0.881	N.S
	8	0.062			9	0.157	N.S		10	0.025	*		11	0.014	*		12	0.034	*
	9	0.014	*		10	0.05	*		11	0.009	**		12	0.021	*		13	0.05	*
	10	0.017	*		11	0.025	*		12	0.014	*		13	0.077			14	0.025	*
	11	0.007	**		12	0.034	*		13	0.101	N.S		14	0.014	*		15	0.034	*
	12	0.186	N.S		13	0.127	N.S		14	0.009	**		15	0.021	*				
	13	0.909	N.S		14	0.025	*		15	0.027	*								
	14	0.123	N.S		15	0.034	*												
	15	0.345	N.S																
	vs	p value			vs	p value			vs	p value			vs	p value					
11	12	0.014	*	12	13	0.48	N.S	13	14	0.655	N.S	14	15	0.624	N.S				
	13	0.053			14	0.624	N.S		15	0.858	N.S								
	14	0.016	*		15	0.564	N.S												
	15	0.027	*																

Figure S8. Combined analysis of 5-HT^{+} serotonergic axons in various groups at Week 12. Data represented as mean ± SD., *p < 0.05; **p < 0.01, Shapiro-Wilk normality test followed by Kruskal-Wallis test and Mann-Whitney post hoc test.

A

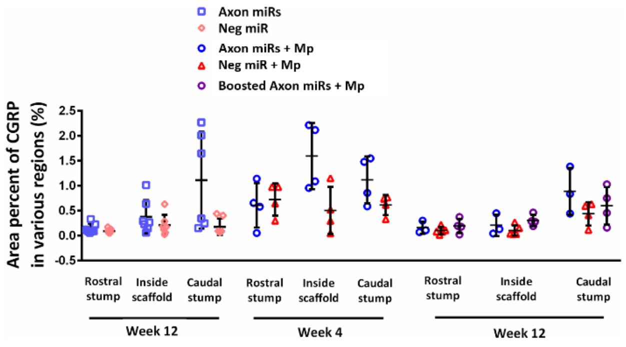

1	Week 12 Axon miRs Rostral
2	Week 12 Neg miR Rostral
3	Week 12 Axon miRs Inside
4	Week 12 Neg miR Inside
5	Week 12 Axon miRs Caudal
6	Week 12 Neg miR Caudal
7	Week 4 Axon miRs + Mp Rostral
8	Week 4 Neg miR + Mp Rostral
9	Week 4 Axon miRs + Mp Inside
10	Week 4 Neg miRs + Mp Inside
11	Week 4 Axon miRs + Mp Caudal
12	Week 4 Neg miR + Mp Caudal
13	Week 12 Axon miRs + Mp Rostral
14	Week 12 Neg miR + Mp Rostral
15	Week 12 Boosted Axon miRs + Mp Rostral
16	Week 12 Axon miRs + Mp Inside
17	Week 12 Neg miRs + Mp Inside
18	Week 12 Boosted Axon miRs + Mp Inside
19	Week 12 Axon miRs + Mp Caudal
20	Week 12 Neg miR + Mp Caudal
21	Week 12 Boosted Axon miRs + Mp Caudal

	vs	p value			vs	p value			vs	p value			vs	p value	
1	2	0.225	N.S	2	3	0.013	*	3	4	0.225	N.S	4	5	0.046	*
	3	0.085			4	0.142	N.S		5	0.153	N.S		6	0.749	N.S
	4	0.665	N.S		5	0.004	**		6	0.225	N.S		7	0.186	N.S
	5	0.01	**		6	0.406	N.S		7	0.45	N.S		8	0.014	*
	6	0.848	N.S		7	0.089			8	0.089			9	0.008	**
	7	0.186	N.S		8	0.008	**		9	0.014	*		10	0.257	N.S
	8	0.014	*		9	0.008	**		10	0.571	N.S		11	0.014	*
	9	0.008	**		10	0.186	N.S		11	0.038	*		12	0.023	*
	10	0.257	N.S		11	0.008	**		12	0.131	N.S		13	0.909	N.S
	11	0.008	**		12	0.008	**		13	0.425	N.S		14	0.291	N.S
	12	0.008	**		13	0.305	N.S		14	0.042	*		15	1	N.S
	13	0.909	N.S		14	0.685	N.S		15	0.345	N.S		16	0.909	N.S
	14	0.291	N.S		15	0.186	N.S		16	0.305	N.S		17	0.291	N.S
	15	0.571	N.S		16	0.569	N.S		17	0.042	*		18	0.131	N.S
	16	0.732	N.S		17	0.465	N.S		18	0.85	N.S		19	0.03	*
	17	0.291	N.S		18	0.008	**		19	0.087			20	0.257	N.S
	18	0.059			19	0.017	*		20	0.705	N.S		21	0.089	
	19	0.017	*		20	0.023	*		21	0.257	N.S				
	20	0.038	*		21	0.014	*								
	21	0.023	*												
	vs	p value			vs	p value			vs	p value			vs	p value	
5	6	0.032	*	6	7	0.186	N.S	7	8	0.773	N.S	8	9	0.083	
	7	0.522	N.S		8	0.023	*		9	0.083			10	0.386	N.S
	8	0.831	N.S		9	0.008	**		10	0.564	N.S		11	0.386	N.S
	9	0.394	N.S		10	0.345	N.S		11	0.149	N.S		12	0.564	N.S
	10	0.394	N.S		11	0.008	**		12	0.773	N.S		13	0.034	*
	11	1	N.S		12	0.023	*		13	0.289	N.S		14	0.014	*
	12	0.831	N.S		13	0.909	N.S		14	0.142	N.S		15	0.043	*
	13	0.071			14	0.808	N.S		15	0.149	N.S		16	0.077	*
	14	0.011	*		15	0.85	N.S		16	0.157	N.S		17	0.014	*
	15	0.136	N.S		16	0.732	N.S		17	0.05	*		18	0.043	*
	16	0.121	N.S		17	0.223	N.S		18	0.248	N.S		19	0.724	N.S
	17	0.028	*		18	0.131	N.S		19	0.48	N.S		20	0.149	N.S
	18	0.394	N.S		19	0.017	*		20	0.564	N.S		21	0.773	N.S
	19	1	N.S		20	0.038	*		21	1	N.S				
	20	0.522	N.S		21	0.023	*								
	21	0.67	N.S												
	vs	p value			vs	p value			vs	p value			vs	p value	
9	10	0.083		10	11	0.083		11	12	0.149	N.S	12	13	0.034	*
	11	0.248	N.S		12	0.386	N.S		13	0.034	*		14	0.014	*
	12	0.021	*		13	0.48	N.S		14	0.014	*		15	0.043	*
	13	0.034	*		14	0.142	N.S		15	0.021	*		16	0.077	
	14	0.014	*		15	0.248	N.S		16	0.034	*		17	0.014	*
	15	0.021	*		16	0.289	N.S		17	0.014	*		18	0.043	*
	16	0.034	*		17	0.05	*		18	0.021	*		19	0.289	N.S
	17	0.014	*		18	0.386	N.S		19	0.289	N.S		20	0.248	N.S
	18	0.021	*		19	0.289	N.S		20	0.083			21	1	N.S
	19	0.157	N.S		20	0.773	N.S		21	0.149	N.S				
	20	0.021	*		21	0.773	N.S								
	21	0.043	*												
	vs	p value			vs	p value			vs	p value			vs	p value	
13	14	0.655	N.S	14	15	0.327	N.S	15	16	1	N.S	16	17	0.297	N.S
	15	0.724	N.S		16	0.456	N.S		17	0.462	N.S		18	0.289	N.S
	16	0.827	N.S		18	0.027	*		18	0.248	N.S		19	0.127	N.S
	17	0.297	N.S		17	0.917	N.S		19	0.034	*		20	0.289	N.S
	18	0.289	N.S		19	0.025	*		20	0.149	N.S		21	0.077	
	19	0.05	*		20	0.027	*		21	0.149	N.S				
	20	0.077			21	0.027	*								
	21	0.077													
	vs	p value			vs	p value			vs	p value			vs	p value	
17	18	0.027	*	18	19	0.077		19	20	0.157	N.S	20	21	0.386	N.S
	19	0.025	*		20	0.386	N.S		21	0.48	N.S				
	20	0.05	*		21	0.386	N.S								
	21	0.027	*												

Figure S9. Combined analysis of $CGRP^+$ sensory axons in various groups at both Week 4 and Week 12. Data represented as mean ± SD., *$p < 0.05$; **$p < 0.01$, Shapiro-Wilk normality test followed by Kruskal-Wallis test and Mann-Whitney post hoc test.

1	Week 12 Axon miRs
2	Week 12 Neg miR
3	Week 4 Axon miRs + Mp
4	Week 4 Neg miR + Mp
5	Week 12 Axon miRs + Mp
6	Week 12 Neg miR + Mp
7	Week 12 Boosted Axon miRs + Mp

A

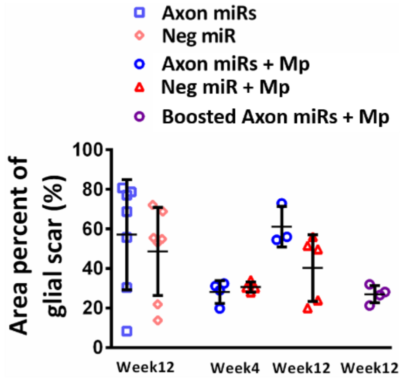

	vs	p value			vs	p value			vs	p value	
1	2	0.277	N.S	2	3	0.186	N.S	3	4	0.773	N.S
	3	0.131	N.S		4	0.257	N.S		5	0.034	*
	4	0.089			5	0.305	N.S		6	0.327	N.S
	5	0.732	N.S		6	0.372	N.S		7	0.564	N.S
	6	0.167	N.S		7	0.186	N.S				
	7	0.089									
	vs	p value			vs	p value			vs	p value	
4	5	0.034	*	5	6	0.053		6	7	0.462	N.S
	6	0.624	N.S		7	0.034	*				
	7	0.248	N.S								

Figure S10. Combined analysis of glial scar in various groups at both Week 4 and Week 12. Data represented as mean ± SD., $*p < 0.05$; $**p < 0.01$, Shapiro-Wilk normality test followed by Kruskal-Wallis test and Mann-Whitney post hoc test.

A

Sample	RIN^e	28S/18S (Area)	Concentration [ng/µl]	Volume [µl]	Yield [µg]
Electronic Ladder	-	-	84.9	NA	NA
SCI_1	8.3	1.0	546	18	9.83
SCI_2	8.6	1.3	342	13	4.45
Neg miR + Mp_1	7.6	1.1	1050	18	18.90
Neg miR + Mp_2	7.9	1.1	1140	18	20.52
Axon miRs + Mp_1	8.4	1.2	532	18	9.58
Axon miRs + Mp_2	7.8	1.1	937	18	16.87
Axon miRs + Mp_3	7.7	0.9	1200	18	21.60
Sham_1	7.6	1.3	1020	10	10.20
Sham_2	7.8	1.2	311	10	3.11
Sham_3	8.3	1.2	241	10	2.41

B

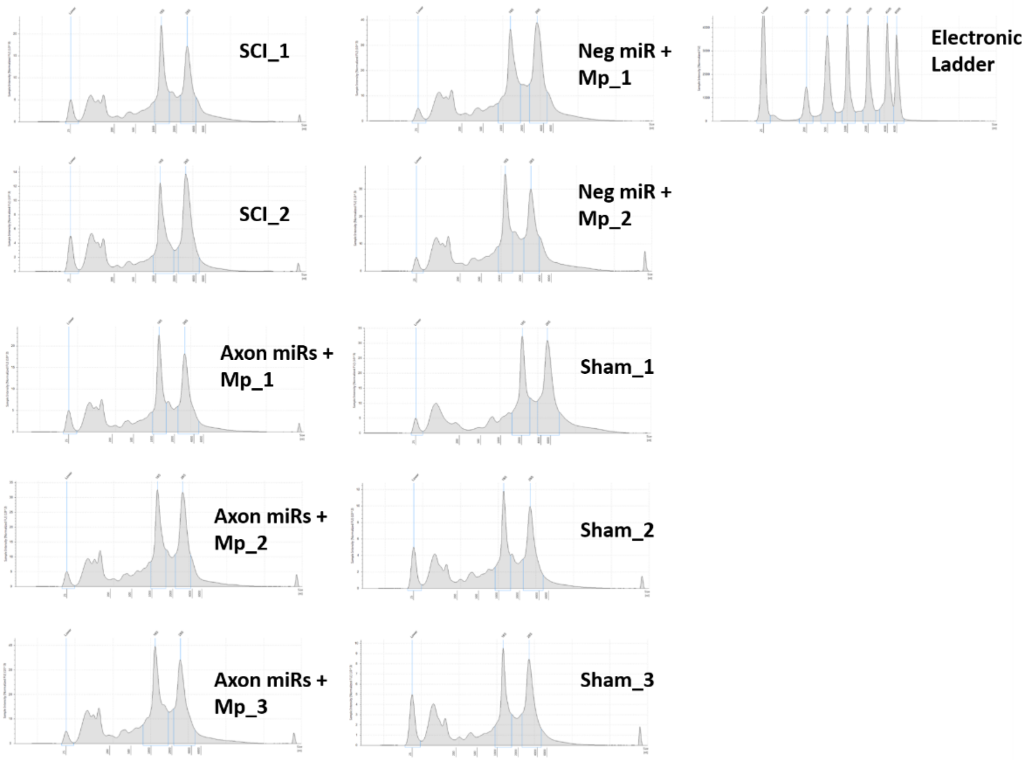

Figure S11. Quality of RNAs used for RNA sequencing. (A) Detailed information of the samples used for RNA sequencing. (B) RNA integrity assay shows two peaks from 18S and 28S, respectively.

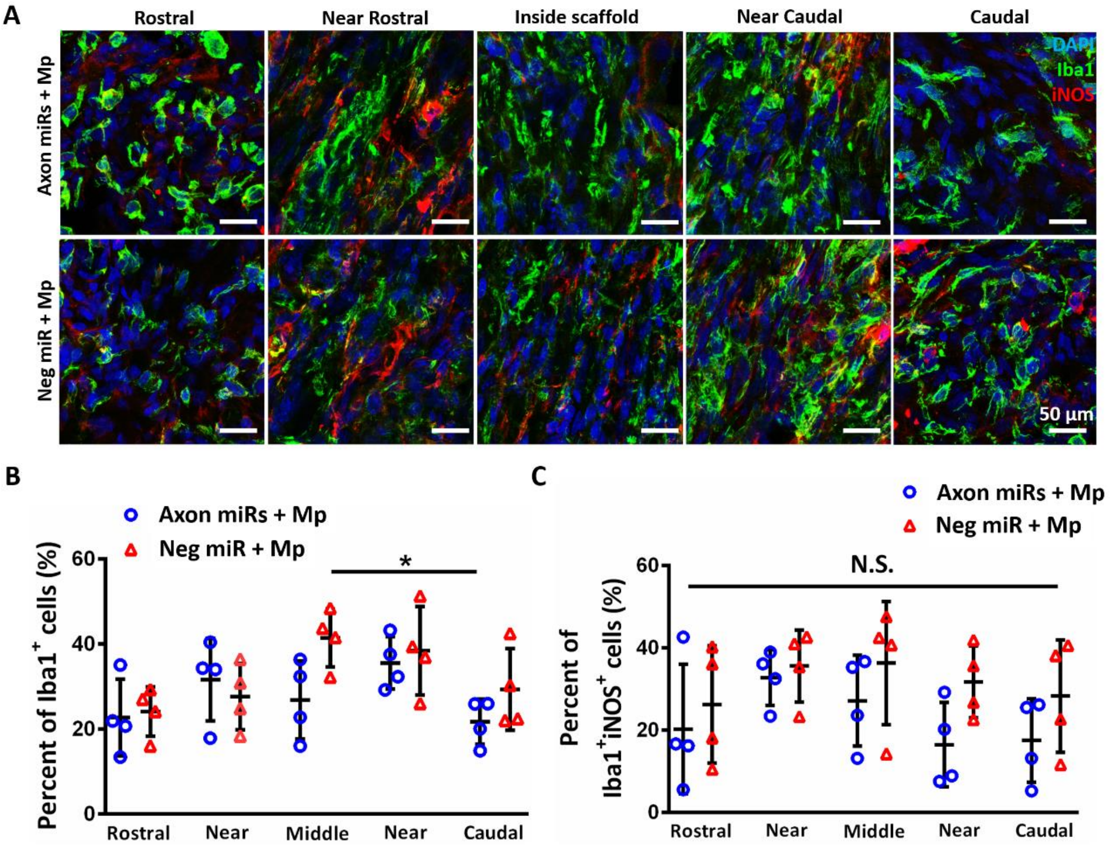

Figure S12. Animals treated with Axon miRs in the presence of methylprednisolone had lower percentage of Iba1^{+}iNOS^{+} cells in injured area. (A) Representative fluorescent images of Iba1 (green) and iNOS (red) expression at rostral, near rostral, inside scaffold, near caudal and caudal regions. (B-C) Quantification of number of Iba-1^{+} cells at different regions suggests there is a trend that the treatment of Neg miR + Mp increased the expression of Iba-1 and iNOS inside scaffold and at caudal regions. However, the difference is not significant. Data represented as mean ± SD., $^{*}p < 0.05$, Shapiro-Wilk normality test followed by Kruskal-Wallis test and Mann-Whitney post hoc test.

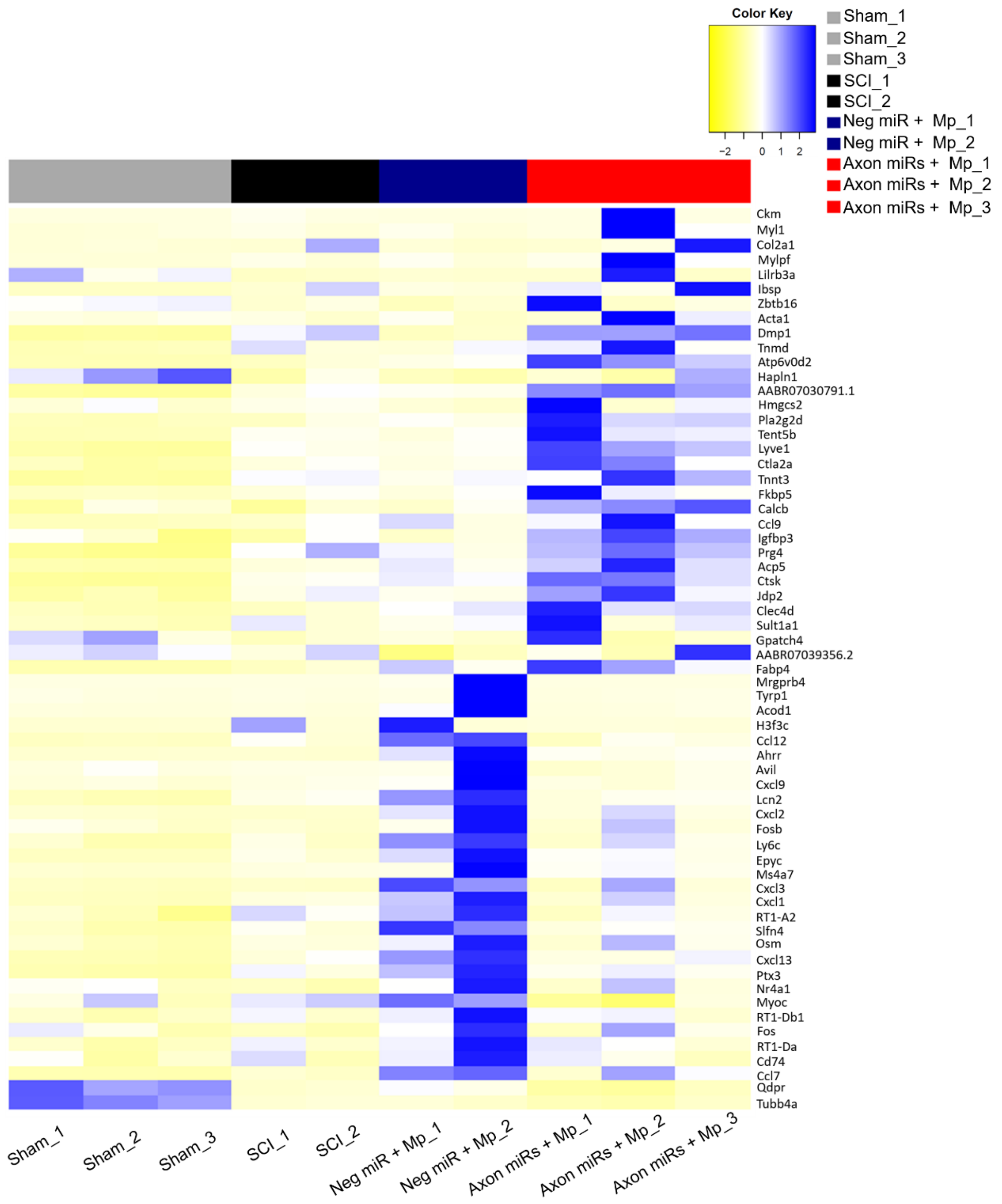

Figure S13. Heatmap of the expression of 62 significant regulated genes in Sham, SCI, Neg miR + Mp and Axon miRs + Mp group.

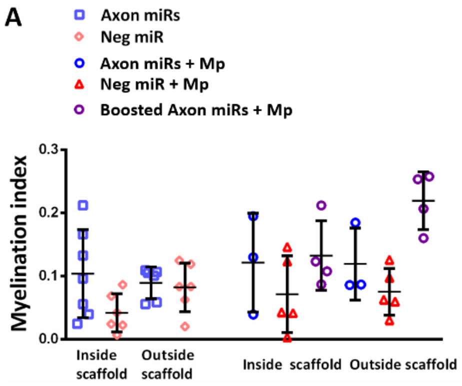

1	Inside scaffold Week 12 Axon miRs
2	Inside scaffold Week 12 Neg miR
3	Outside scaffold Week 12 Axon miRs
4	Outside scaffold Week 12 Neg miR
5	Inside scaffold Week 12 Axon miRs + Mp
6	Inside scaffold Week 12 Neg miR + Mp
7	Inside scaffold week 12 Boosted Axon miRs + Mp
8	Outside scaffold Week 12 Axon miRs + Mp
9	Outside scaffold Week 12 Neg miR + Mp
10	Outside scaffold Week 12 Boosted Axon miRs + Mp

	vs	p value			vs	p value			vs				vs	p value	
1	2	0.063		2	3	0.025	*	3	4	1	N.S	4	5	0.302	N.S
	3	0.886	N.S		4	0.15	N.S		5	0.439	N.S		6	0.715	N.S
	4	0.568	N.S		5	0.121	N.S		6	0.584	N.S		7	0.136	N.S
	5	0.909	N.S		6	0.465	N.S		7	0.136	N.S		8	0.197	N.S
	6	0.465	N.S		7	0.011	*		8	0.796	N.S		9	0.855	N.S
	7	0.45	N.S		8	0.039	*		9	0.584	N.S		10	0.011	*
	8	0.732	N.S		9	0.144	N.S		10	0.011	*				
	9	0.685	N.S		10	0.011	*								
	10	0.038	*												
	vs	p value			vs	p value			vs	p value			vs	p value	
5	6	0.456	N.S	6	7	0.327	N.S	7	8	0.289	N.S	8	9	0.297	N.S
	7	1	N.S		8	0.297	N.S		9	0.142	N.s		10	0.077	N.S
	8	0.827	N.S		9	0.754	N.S		10	0.083					
	9	0.297	N.S		10	0.014	*								
	10	0.077													
	vs	p value													
9	10	0.014	*												

Figure S14. Combined analysis of myelination index in different regions at Week 12. Data represented as mean ± SD., *$p < 0.05$; **$p < 0.01$, Shapiro-Wilk normality test followed by Kruskal-Wallis test and Mann-Whitney post hoc test.

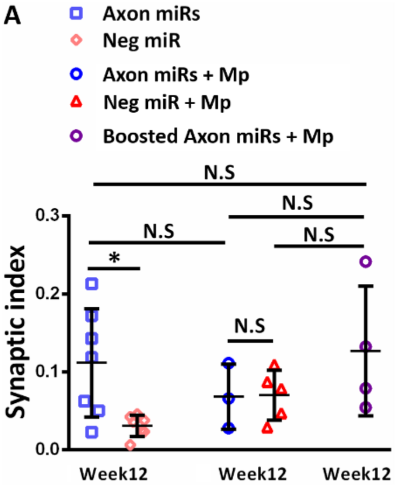

1	Week 12 Axon miRs
2	Week 12 Neg miR
3	Week 12 Axon miRs + Mp
4	Week 12 Neg miR + Mp
5	Week 12 Booste Axon miRs + Mp

	vs	p value			vs	p value			vs	p value	
1	2	0.018	*	2	3	0.138	N.S	3	4	0.881	N.S
	3	0.452	N.S		4	0.028	*		5	0.289	N.S
	4	0.291	N.S		5	0.008	**				
	5	0.705	N.S								
	vs	p value									
4	5	0.221	N.S								

Figure S15. Combined analysis of synaptic index in various groups at Week 12. Data represented as mean ± SD., *p < 0.05; **p < 0.01, Shapiro-Wilk normality test followed by Kruskal-Wallis test and Mann-Whitney post hoc test.
